# Addressing gender bias in sexual and reproductive health care: A scoping review of training tools and interventions

**DOI:** 10.1371/journal.pone.0359704

**Published:** 2026-10-13

**Authors:** Chioma Oringanje, Joshua Meremikwu, Babasola Okusanya, Chukwudi Moses Oringanje, Moriam Chibuzor, Ememobong Aquaisua, Anthony Okoro, Deborah Ndukwu, Martin Madu Meremikwu

**Affiliations:** 1 Department of Biology, Xavier University, Cincinnati, Ohio, United States of America; 2 Cochrane Nigeria, Institute of Tropical Diseases Research and Prevention, University of Calabar Teaching Hospital, Calabar, Nigeria; 3 Department of Obstetrics and Gynecology, College of Medicine, University of Lagos Idi-Araba, Lagos, Nigeria; 4 Department of Computer Science, Faculty of Computing, University of Calabar, Calabar, Nigeria; 5 Syndicate Bio, Lagos, Nigeria; 6 Department of Paediatrics, University of Calabar Teaching Hospital, Calabar, Nigeria; Universidad Peruana Cayetano Heredia Facultad de Medicina, PERU

## Abstract

Gender norms and bias influence how health workers interpret and deliver sexual and reproductive health and rights (SRHR) services, contributing to stigma, discrimination, and inequities in care. This scoping review aimed to map training tools and educational interventions designed to strengthen health workers’ competencies and address gender bias in SRHR care. This scoping review followed the Arksey and O’Malley framework and PRISMA-ScR guidelines. Searches were conducted across multiple databases and organizational websites for literature published between 2000 and November, 2025. Eligible records included manuals, curricula, toolkits, and educational interventions targeting health workers and addressing gender norms, bias, values, or stigma in SRHR care. Data were extracted and synthesized using descriptive and narrative approaches. A total of 128 sources were included, comprising 25 toolkits/manuals and 103 intervention reports representing 100 unique intervention approaches. Together, they covered diverse SRHR domains and used multi-component pedagogical approaches, including didactic instruction (98.4%), reflective or values-based learning (83.2%), and experiential methods such as role-play, simulation, and case-based learning (92%). Most interventions emphasized equitable, person-centered care (98.4%) and 68.8% incorporated gender-transformative elements, while 74.4% included system-level components. **Across the mapped literature, gender-transformative and system-level approaches were incorporated to varying degrees, reflecting variability in their integration across educational interventions. Overall, the review highlights the breadth and characteristics of existing training approaches and identifies opportunities for greater integration of gender-transformative and system-level strategies in future** SRHR **training and evaluation.**

## Introduction

Gender norms are the spoken and unspoken rules of societies that determine which attributes and behaviors are valued and considered acceptable for men, women, and gender minorities [1,2]. These norms, particularly those related to sexuality, independence, and bodily autonomy, shape the behaviors, roles, and traits considered appropriate for women and girls as well as for men and boys, and often begin to exert influence over individuals even before their birth [3,4]. Gender norms significantly impact health across the life course and interact with other social determinants of health across contexts. Early social influences shape health outcomes differently for girls and boys, and deviation from socially prescribed norms can result in adverse health consequences, particularly when accompanied by stigma or discrimination [2]. These norms are deeply embedded within social institutions and organizations, including healthcare systems, where they are reinforced through everyday interactions.

Gender bias remains pervasive worldwide and continues to shape opportunities, wellbeing, and health outcomes across the life course. The 2023 United Nations Development Programme (UNDP) Gender Social Norms Index found that nearly nine out of ten people hold at least one bias against women [5]. This indicates that discriminatory gender norms remain prevalent across political, educational, economic, and bodily autonomy domains. These deeply entrenched social norms influence access to resources, decision-making power, and healthcare utilization, contributing to inequities in health outcomes across populations. Health systems not only deliver care but also reflect and reproduce societal norms and power structures, including gender norms that influence service delivery, workforce dynamics, financing, and governance [6,7]. Within these systems, healthcare workers may consciously or unconsciously perpetuate gender stereotypes, resulting in unequal treatment of patients with similar conditions and discrimination against individuals who do not conform to traditional gender expectations.

Access to quality sexual and reproductive health (SRH) services is essential for safeguarding the sexual and reproductive rights and wellbeing of all individuals. However, stigma, bias, and discrimination within healthcare systems can create barriers to equitable care at structural, interpersonal, and individual levels. Stigma stems from negative societal perceptions, bias reflects unequal treatment, and discrimination results in unfair actions that marginalize individuals who diverge from conventional gender norms [4]. Over time, gender-based discrimination may become embodied, contributing to chronic stress, social exclusion, and poorer health outcomes [1]. Healthcare workers’ personal and cultural beliefs about gender can influence their attitudes, communication, clinical assessment, and decision-making in SRH care settings. Implicit gender biases may affect care quality, compromise patient rights, and exacerbate health disparities. Recognizing this, several organizations, including the World Health Organization (WHO), have emphasized the importance of addressing gender norms and biases within healthcare systems [8].

Various competency-based interventions and training approaches in sexual and reproductive health and rights (SRHR) have been developed to strengthen health workers’ competencies and align clinical practice with SRHR principles. These approaches commonly include values clarification exercises, simulation, reflective practice, role-plays, guided learning, mentorship, shared decision-making (SDM), and communication, and respectful, person-centered care training [9–11]. Many interventions engage health workers in examining their own and collective values, beliefs, attitudes, and biases that shape interactions with clients and influence care behaviors [12]. Some interventions are also informed by critical and feminist pedagogical approaches that use storytelling, reflection, and facilitated dialogue to encourage examination of gender, power, and agency within healthcare practice. In addition to individual-level competency development, some initiatives incorporate facility- or system-level strategies such as strengthening respectful maternity care policies and improving communication practices to support more respectful and person-centered care [9,13].

The spectrum and characteristics of tools and approaches designed to strengthen healthcare workers’ competencies and address gender bias in SRH care have not been comprehensively described in spite of the attention gender bias in healthcare has garnered. Therefore, the objectives of this scoping review are to identify and summarize the types of tools and educational approaches available to strengthen health workers’ competencies for addressing gender bias in sexual and reproductive health (SRH) care, including value clarification and other strategies that promote equitable, respectful, and rights-based care.

## Methods

### Study design

This scoping review was conducted using the Arksey and O’Malley methodological framework, which consists of five phases: identifying the research question, identifying relevant studies, study selection, charting the data, and collating, summarizing, and reporting the results [14]. The Preferred Reporting Items for Systematic Reviews and Meta-Analysis Extension for Scoping Reviews (PRISMA-ScR) was used for the scoping review protocol preparation and report presentation [15]. The review was registered in the Open Science Framework (https://doi.org/10.17605/OSF.IO/S5M84).

### Search strategy

A comprehensive search strategy was developed and applied across multiple electronic databases, including MEDLINE (Ovid), CINAHL, Embase (Ovid), CENTRAL (Ovid), ERIC (Ovid), PsycINFO (Ovid), and Google Scholar, covering the period from January 01, 2000 to November 25, 2025. To ensure inclusion of materials not indexed in biomedical databases and not published in commercial journals, we also searched organizational websites (WHO, UNFPA, IPPF, JHPIEGO, IPAS, PATH, and EngenderHealth) and additional grey literature sources as many relevant tools and interventions are produced by these organizations. Search strategies were tailored to each database and website. Records retrieved from bibliographic databases were imported into Covidence® for duplicate removal and title/abstract screening. Records identified through organizational website searches were not imported into Covidence. These were screened manually using the same eligibility criteria applied to database records, with discrepancies resolved through team discussion. For Google Scholar, 800 records were initially retrieved using four separate search strings, each retrieving up to 200 records. Following preliminary deduplication in EndNote, 124 duplicate records were removed before import into Covidence, leaving 676 records for title and abstract screening [16]. The full search strategy is presented in Supplementary 1 in S1 Data.

### Eligibility criteria

This review included studies that focused on healthcare workers, defined as any cadre providing direct clinical care, such as nurses, midwives, physicians, clinical officers, nursing students, medical residents, and community health workers. Eligible studies described or evaluated training materials, curricula, toolkits, or educational approaches aimed at strengthening competencies related to gender norms, gender bias, or respectful, rights-based sexual and reproductive health (SRH) care. Eligibility required the presence of a defined training or educational component with sufficient detail to understand its content, purpose, and mode of delivery. Interventions were eligible if they supported health workers in recognizing or challenging underlying beliefs, norms, or biases that influence SRH service provision, or otherwise strengthened competencies to provide equitable, person-centered, or rights-based SRH care through educational strategies such as didactic instruction, experiential learning, reflective or values-based approaches, communication skills or systems-oriented training, or combinations of these approaches.

Studies were excluded if they did not involve healthcare workers, did not describe a training material, curriculum, or educational approach, focused solely on service delivery or clinical outcomes without an educational or capacity-building component, or lacked relevance to gender, bias, values, or sexual and reproductive health and rights (SRHR). Studies that mentioned gender, equity, attitude, or values without describing an educational approach aimed at strengthening healthcare workers’ competencies for SRHR care were excluded. In line with prespecified scope of this review, we excluded studies with a primary focus on HIV-related training because this topic has been extensively researched and falls outside the primary focus of this review. There were no language, study design, or geographic setting restrictions.

### Study selection

All records identified through database searches and grey literature sources were imported into Covidence® [16]. Duplicate records were removed. Records from organizational websites were screened manually. The screening was conducted in two stages. First, titles and abstracts were independently reviewed by two reviewers to assess potential eligibility. Records that appeared to meet the inclusion criteria or for which eligibility was unclear were advanced to full-text review. In the second stage, full-text reports were independently assessed by two reviewers to determine final inclusion, and when included, data extraction was performed. Discrepancies at either stage were resolved through discussions or consensus, with consultation with a third reviewer when necessary. Reasons for exclusion at the full-text stage were documented and are presented in Supplementary 2 in S1 Data. The selection process is summarized in a PRISMA flow diagram (see Fig 1).

**Fig 1:**
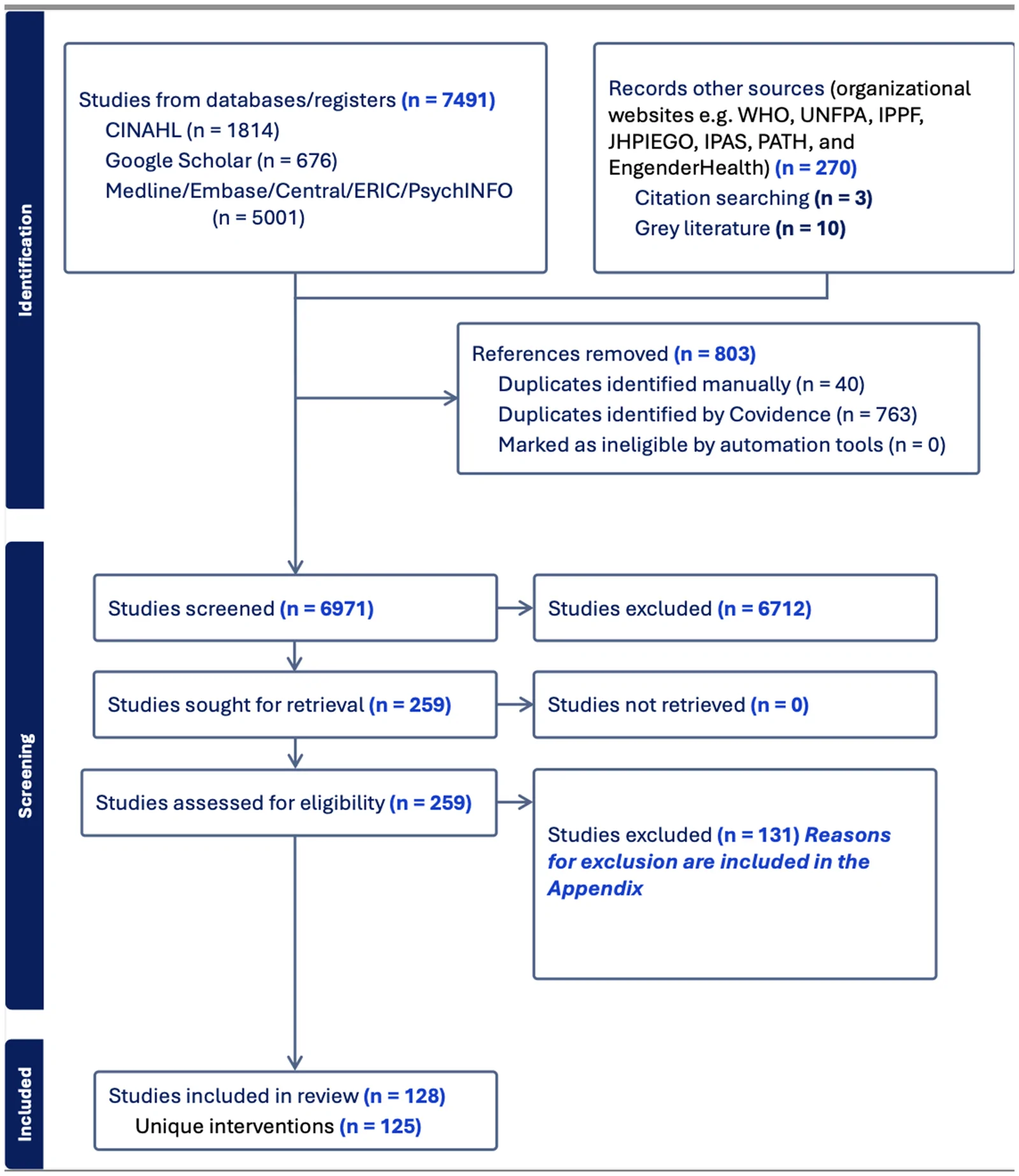
Scoping review PRISMA flowchart.

### Data charting

A standardized data-charting Excel sheet was developed for data extraction. The form was piloted on a subset of included studies and refined iteratively through team discussion. Two reviewers independently extracted data, including citation details (authors, year, title, source), study location, type of publication (e.g., peer-reviewed article, grey literature, training manual), target population (e.g., health worker cadre), objectives, and description of the intervention or tool. Discrepancies were resolved through discussion and consensus within the review team.

In addition, detailed information was extracted on training characteristics, including content domains (e.g., respectful maternity care, gender-based violence, abortion care, LGBTQ+ health), pedagogical approaches (e.g., values clarification and attitude transformation, simulation or role-play, case-based learning, reflective practice, didactic instruction, digital or online learning, mentorship or quality improvement approaches), delivery modality (in-person, online, or blended), target level of influence, and duration where reported.

For the purposes of synthesis, interventions and tools were coded using a structured analytical framework and codebook developed for this review and applied consistently across all included sources. Binary variables were coded as present (1) or absent (0) based on explicit descriptions in the source. Where classifications (e.g., gender-transformative elements, delivery modality, content domains, pedagogical approaches, transformative focus, or level of influence) required interpretation, coding was based on the stated objectives, curriculum content, and intervention activities reported in each source. Ambiguous cases were resolved through discussion among reviewers until consensus was reached.

Sources were grouped according to (i) content domain, (ii) pedagogical approach, and (iii) level of influence. Content domains were defined as the primary thematic focus of the training (e.g., gender-based violence, abortion care), and pedagogical approaches as the instructional methods used (e.g., role-play, case-based learning). Level of influence was defined as the primary level at which the intervention aimed to effect change: individual (targeting provider knowledge, attitudes, values, skills, or behaviors), interpersonal (targeting provider-patient communication, interactions, relationships, or empathy), or structural (targeting organizational, institutional policy, leadership, accountability, service delivery, or broader health system factors).

Interventions were considered to incorporate **gender-transformative elements** when they explicitly sought to challenge or change harmful gender norms, unequal gender power relations, discrimination, or structural gender inequalities within SRHR care or health systems. Coding reflected the intervention’s underlying approach or theory of change rather than its primary topic. Consequently, an intervention could be classified as gender-transformative even when its primary content domain was another SRHR topic (e.g., gender-based violence, abortion care, family planning, respectful maternity care, or adolescent SRHR), provided it explicitly aimed to transform gender norms, power relations, or structural inequalities. Because interventions frequently operated across multiple levels, more than one target-level code was assigned where appropriate. Similarly, multiple codes were assigned when interventions addressed more than one content domain or pedagogical approach. Detailed coding definitions and decision rules are provided in **Supplementary Codebook (Supplementary 3 in S1 Data)**, and the complete source-level coding matrix is available in **Supplementary 4A and 4B in S1 Data**.

Data were synthesized descriptively using frequencies and proportions, and narratively to identify patterns across studies. Interventions and tools were grouped and analyzed according to key groupings. Where more than one publication described the same intervention or training tool, these were grouped and treated as a single unit of analysis to avoid double counting. Similarly, related materials (e.g., trainer and participant manuals) were consolidated and reported as a single tool where appropriate.

A formal critical appraisal of included sources of evidence was not conducted, as the aim of this scoping review was to map the range and characteristics of available evidence rather than to assess methodological quality or risk of bias.

## Results

The search yielded 7774 records, comprising 7491 records from bibliographic databases searching, 270 records from organizational websites, 3 records identified through citation searching, and 10 records from grey literature. After removal of 803 duplicate records, 6971 records remained for title and abstract screening. Following screening, 259 full-text articles were assessed for eligibility. A total of 128 publications were included in the review, comprising103 intervention publications and 25 training tools (toolkits/manuals). Three intervention publications described interventions already reported in companion publications; therefore, 103 publications represented 100 unique educational intervention approaches. Together with the 25 training tools, the review synthesized 125 analytical units.

The 25 included toolkits/manuals (Table 1) spanned diverse geographical contexts and were primarily developed by international organizations, ministries of health, and non-governmental organizations. Most were designed for global use (18/25, 72%), while the remainder (7/25, 28%) were developed for specific national or regional contexts. The 100 included interventions (Table 2) were implemented across multiple regions, with the highest representation in North America (33/100, 33%), followed by Africa (25/100, 25%) and Asia (18/100, 18%). Smaller proportions were reported for Europe (6/100, 6%), Europe/Asia (5/100, 5%), and Oceania (5/100, 5%). A small number of interventions were conducted across multi-country regions (3/100, 3%), South America (2/100, 2%), Latin America (1/100, 1%), or did not report a specific location (2/100, 2%).

**Table 1:** Characteristics of 25 included manuals/curricula/toolkits (Listed alphabetically by title).

Title of Toolkit / Manual	Organization (year)	Geographic focus	Objective	Target population/ Intended Audience	Key Content/Description^a^
*Abortion Attitude: Transformation Values Clarification Activities Adapted for Young Women*	Ipas 2008a [17]	Global	Designed to help participants critically examine their values and attitudes toward abortion and reduce stigma. Excerpt: “A values clarification toolkit… designed to effectively facilitate abortion values clarification and attitude transformation (VCAT) interventions.”	Trainers, program managers, and technical advisors facilitating SRHR/abortion trainings. Excerpt: “For trainers, program managers and technical advisors who organize or facilitate training events…”	Includes structured VCAT exercises, reflection activities, group discussions, and worksheets to challenge beliefs, stigma, and norms around abortion. Excerpt: “VCAT interventions help participants examine their attitudes and understand how values influence abortion care and access.”
Abortion Attitude Transformation: A Values Clarification Toolkit for Global Audiences	Ipas 2008b [18]	Global	Use values clarification and attitude transformation (VCAT) to shift beliefs toward support for comprehensive, woman-centered abortion care.	Health-care providers and public health professionals (primary audience) but Policymakers, lawyers, legal professionals, advocates, donors, media, religious leaders, community/laypersons.	Values clarification activities; theoretical framework; facilitator guidance; 14 + workshop activities; surveys; agendas; second-trimester abortion modules; additional resources
*Abortion Attitude Transformation: A Values Clarification Toolkit for Humanitarian Audiences*	Ipas 2018a [19]	Global	Reduce abortion stigma and increase provider comfort and effectiveness in humanitarian SRHR settings	Humanitarian health staff, including clinical providers in crisis settings.	Values Clarification & Attitude Transformation; abortion-related stigma, myths, and knowledge gap
Abortion Care for Young Women: A Training Toolkit	Ipas 2011a [20]	Global	Ensure abortion programs are “accessible to and appropriate for young women.”	Abortion providers, trainers, managers.	Youth-friendly abortion care, legal/policy barriers, bias reflection, counseling skills, case studies. “Provides information and guidance on delivering… abortion care for young women (10–24).”
ABORTION STIGMA ENDS HERE*: A toolkit for understanding and action*	IPAS – 2018b [21]	Global	Recognizing, understanding, and reducing abortion stigma and improving respectful, rights-based care	Community-based organization (CBO) and NGO staff/members; community health workers	Abortion stigma framework; structured reflection; values clarification; participatory dialogue; stigma mapping; power & gender analysis; inclusive language; rights-based framing; action planning; community & provider engagement
*Addressing violence against women in pre-service health training*	WHO 2022 [11]	Global	seeks to provide pre- service health training programmes, such as in medical, nursing and midwifery schools, with the knowledge and resources to better prepare their students to care for women subjected to violence.	designed primarily for pre-service medical, nursing and midwifery training programmes, as well as other health worker trainees	builds competencies for health workers to recognize and respond to violence against women by fostering self-awareness of biases, enhancing empathic and non-judgmental communication, and teaching first-line and clinical care skills. It also trains providers to identify signs of abuse, deliver supportive, survivor-centered treatment, and coordinate effectively with multidisciplinary services.
*Adolescent and youth health for healthcare service providers (Participant’s manual)*	Federal Ministry of Health, Ethiopia 2017 [22]	Ethiopia	Orient and sensitize healthcare managers and providers to particular features of adolescent and youth that layout appropriate approaches to properly and adequately respond to selected priority health needs and problems of adolescents.	Physicians, nurses, HEWs, counselors	14 chapters covering SRH, GBV, ethics, development, communication, MI, NCDs, nutrition, HIV,
Caring for women subjected to violence: Online facilitated training for health workers	WHO 2025 [23]	Global	To strengthen provider capacity to recognize, respond to, and support women experiencing violence.	trainers/ facilitators and health worker	Understanding violence against women and survivor experience; Guiding principles & communication; Identifying Intimate partner violence; First-line support (LIVES): Listen, Inquire, Validate Enhance safety and support
Counseling for Effective Use of Family Planning: Trainer’s Manual	EngenderHealth / ACQUIRE Project – 2008 [24]	Global	*aims to improve the knowledge, skills, and attitudes of trainees in assessing and addressing clients’ FP needs through individualized counseling.*	wide range of healthcare workers (physicians, nurses, counselors, technicians, supervisors, frontline staff, outreach workers)	Describe the roles of providers and other health care staff in supporting clients’ informed and voluntary decision making; Encourage creating client’s profiles that create “client profiles” that reflect the unique SRH situation and challenge providers’ stereotypes, biases, and misconceptions
*Disability Inclusion in Reproductive Health Programs – VCAT Toolkit (VCATDS2E21b)*	Ipas 2021 [25]	Global	Designed to integrate disability-inclusion principles into SRHR and abortion work by challenging stigma and bias.	Facilitators who train humanitarian and SRHR workers, including clinical and non-clinical staff.	Contains values clarification activities, disability-inclusive SRHR sessions, stigma-reduction modules, reflective discussions, and exercises addressing abortion stigma and disability discrimination. Excerpt: “Activities allow participants to critically examine how stigma, discrimination and exclusion affect abortion and SRHR for people with disabilities.”
*Effective Training in Reproductive Health: Course Design and Delivery (Reference & Trainer’s Manual)*	IPAS 2011b [26]	Global	Capacity building; competency-based training; adult learning; values clarification; performance improvement; trainer development	Trainers, health-care providers, managers	Adult learning principles; participatory methods; values clarification exercises; facilitation & communication skills; role play; coaching; competency-based assessment; evaluation tools; action planning; transfer of learning
Engaging Boys and Men in GBV Prevention and Reproductive Health in Conflict and Emergency-Response Settings	ACQUIRE Project (EngenderHealth) – 2008 [27]	Global	help participants understand the importance of engaging men and boys in GBV prevention and reproductive health during conflict and emergencies, explore how crises influence gender dynamics and opportunities for male engagement, and develop concrete action steps for integrating these approaches into their existing program workplans.	Broad audience which includes health care provider (Specific audiences: NGO project managers, field staff, health sector coordinators, health promoters, donor representatives, local representatives of ministries of health, and community liaisons working for UNCHR or other U.N. agencies.)	covers gender, GBV, and reproductive health concepts in conflict settings, teaching participants how crises shape gender dynamics and how to positively engage men and boys as clients, partners, and agents of change. It includes practical frameworks, participatory activities, and action-planning steps to integrate male-engagement strategies into GBV prevention and RH programs.
Facilitator’s Guide: Training Manual for Medical Officers	Ministry of Health & Family Welfare (India) – 2014 [28]	India	Build Medical Officer skills for adolescent-friendly SRHR.	Medical Officers providing adolescent care.	Modules on contraception, unsafe abortion, SRHR concerns, communication, mental health, violence. “Module VII – Pregnancy and Unsafe Abortions in Adolescents.”
IMPLEMENT: Health System Response to Gender-Based Violence: Training Manuals on GBV for Health Professionals	Austrian Women’s Shelter Network 2015 [29]	European region	aim to assess health system and legal gaps in providing obstetric and emergency care to GBV survivors, and to strengthen national capacity by training healthcare professionals and advocates to deliver improved, legally supported, victim-centered services. They also seek to promote policy-level advocacy, enhance referral networks, and disseminate best practices across the EU to drive institutional change.	GBV advocates who provide frontline assistance to victims; obstetric/emergency health professionals within clinical teams who are not equipped to as- sist victims of GBV with their specialized needs; management and front-line staff; and health sector policy makers	covers the key pillars of the Global Strategy: survive, thrive, and transform by building health-care providers’ competencies in delivering equitable, respectful, rights-based care for women, children, and adolescents. It includes modules on quality of care, gender and equity, adolescent-responsive services, accountability, multisectoral collaboration, and practical implementation strategies to strengthen SRHR services and reduce discriminatory or harmful provider practices.
Integrating Gender-Based Violence Response into FP & RH Service Delivery: Trainer’s Manual	Pathfinder International; Directorate General of Family Planning 2023 [30]	Bangladesh	*to advance the knowledge and skill set of FP and SRH service providers to address GBV and provide GBV-responsive care.*	FP providers (nurses, midwives, Female welfare visitors).	Gender, values/bias, GBV types, survivor-centered FP counseling, understanding gender, GBV, gender norms, gender equity, gender and power, male engagement, Legality, Protecting Confidentiality, and Reporting GBV
*Person-centered communication for female genital mutilation prevention: A facilitator’s guide for training health-care providers*	WHO 2022 [31]	Global	“The overall aim of this training is to strengthen the knowledge and skills of health-care providers to be active agents of change for the prevention of FGM, including to challenge their own values…”. “Participants will explore their values towards FGM… and learn ways to resist requests to do FGM.”	Health-care providers with basic clinical and counselling skills.	understanding of traditions, values, and social norms driving FGM; builds their knowledge of the health consequences and ethical issues; and strengthens their skills in person-centered communication to prevent FGM, challenge myths, and respond ethically to requests for medicalized cutting. Through participatory activities, values-clarification exercises, role-plays, and discussions, providers learn to influence community change, uphold professional ethics, and promote the abandonment of FGM.
*Postabortion Care Curriculum – Participant Guide & Reference Manual*	EngenderHealth 2024 [32]	Global	To build competency-based clinical and counseling skills for safe, rights-based postabortion care, including values and attitudes, clinical management, counseling, FP, respectful care, and community linkages.	Skilled healthcare personnel including “midwives, nurses, clinical officers… and physicians	25 structured clinical and non-clinical training sessions that strengthen providers’ competencies in values and attitudes, postabortion clinical management, uterine evacuation skills, FP counseling, infection prevention, and person-centered, youth-friendly care. Learning is reinforced through participatory methods including role-plays, case studies, skills practice, and scenario-based exercises.
Promoting the Health of Men Who Have Sex With Men Worldwide: A Training Curriculum for Providers	MSMGF + Johns Hopkins University 2014 [33]	Global	*aims to provide local community groups serving gay men and other MSM with the ability to build cultural and clinical competency amonghealthcare providers serving their communities*	Healthcare providers serving MSM.	nine-module training designed to build healthcare providers’ cultural competence, communication skills, and clinical capacity to deliver respectful, evidence-based, and non-discriminatory care to gay men and other MSM. It covers sexuality, mental health, barriers to care, HIV/STI prevention and treatment, and practical strategies for creating inclusive, rights-affirming health services.
Providers as Advocates for Safe Abortion Care: Training Manual	Ipas 2009 [34]	Global	*to help providers understand their power and role as advocates, develop an advocacy mindset, and recognize when and how to effectively advocate for comprehensive abortion care within the full scope of the law*	Health-care providers who are involved in delivering abortion and/or postabortion care	understanding advocacy, recognizing and using their personal and political power, and applying these skills to advocate for safe abortion care within legal frameworks. It combines foundational concepts, interactive activities, and practical planning to help providers identify their influence, expand their advocacy roles, and take concrete action.
REDI: Client-Centered Counseling Framework – Trainer’s Manual/Participants Handbook	EngenderHealth 2018 [35]	Global	Improve FP counseling through rapport, exploration, decision-making, implementation.	Healthcare service providers, their supervisors, and managers of the programs	a comprehensive, rights-based, client-centered counseling curriculum covering SRHR principles, client decision-making, provider attitudes, communication skills, and the full REDI counseling framework. It guides providers through practical sessions on exploring client needs, supporting decisions, addressing misconceptions, and tailoring counseling for diverse client groups, including adolescents, couples, and clients facing IPV.
Reproductive Health Training Module for In-service Training. Module II: Comprehensive Family Planning Facilitator Guide	Ministry of Health Liberia (with USAID) 2019 [36]	Liberia	*“designed for use as part of thecomprehensive family planning (FP) and reproductive health (RH) training of physicians, nurses, midwivesand physician assistant.”*	Physicians, nurses, midwives, physician assistants. “…training of physicians, nurses, midwives and physician assistant.”	Covers: Reproductive health, adolescent health, male involvement, counseling, contraceptive method provision, infection prevention. Includes simulations, clinical skills practice, OSCEs, cue cards. “Sessions include simulation skills practice, discussions, clinical practice… skills checklists, and counseling cue cards.”
*Respectful Maternity Care: Operational Guidance for Providers (Orientation Package)*	Afghanistan Ministry of Public Health 2017 [37]	Afghanistan	build participants’ understanding of respectful maternity care—its current context, key concepts, rights-based frameworks, VCAT approaches, and evidence-based strategies to reduce mistreatment—while equipping them to promote RMC and develop actionable plans for implementing it at multiple levels.	Skilled birth attendants, midwives, nurses, doctors.	overview of respectful maternity care, covering women’s childbearing rights, mistreatment categories, and a rights-based legal framework; explores provider self-awareness, stress, and debriefing as factors influencing behavior; reviews ethics, codes of conduct, and professional responsibilities; and emphasizes mutual accountability and management-level strategies to promote RMC. Included a VCAT module, 3-day HCW training schedule, respectful maternity care practices, communication, empathy exercises.
Sexual pleasure: The forgotten link in sexual andreproductive health and rights	Global Advisory Board (GAB) for Sexual Health and Wellbeing 2018 [38]	Global	equips trainers to strengthen health professionals’ ability to deliver high-quality, rights-based SRH services using GAB’s integrated approach to sexual health, rights, and pleasure. It also guides providers to examine their own values, opinions, and prejudices to enhance respectful, client-centered care.	Medical students, public-health professionals, social workers, psychologists, existing health providers	centered on sexual health and rights, including gender norms, values clarification, equity, consent, and positive sexuality. It challenges providers’ assumptions and promotes reflective learning on gender, pleasure, and stigma, particularly as they affect marginalized groups such as LGBTQI+ individuals and youth.
Transforming health systems: gender and rights in reproductive health: a training curriculum for health programme managers	World Health Organization (WHO) 2001 [39]	Global	increase the number of healthcare workers who understand a gender and rights perspective on health, and who have the analytical tools to help implement gender and rights-sensitive reproductive health policies and programme	health trainers to use with health managers, planners, policymakers and others with responsibilities in reproductive health	session-based and case-based curriculum on how to promote gender equity and reproductive rights through the use of evidence, policy development and service delivery.
Youth Friendly Services for Sexual andReproductive Health: Facilitators’ Guide for Training of Providers	UNFPA (United Nations Population Fund) 2001 [40]	India	to improve the quality and youth-friendliness of SRH service delivery	Healthcare providers	Contents include training healthcare providers in youth-friendly, rights-based SRH service delivery, covering clinical skills, counseling techniques, and adolescent health needs, alongside strong values-clarification exercises to reduce bias and ensure non-judgmental, respectful care.

^a^ *Content domains were coded as non-mutually exclusive categories. Individual toolkits could address multiple domains; therefore, percentages do not sum to 100%.*

**Table 2:** Characteristics of the 100 Included interventions (Listed alphabetically by Study ID).

Study ID	Country/Setting	Intervention/Training Material	Target participants	Objective/Focus	Key Content	Delivery & Pedagogical Approach
**Afulani (2019, 2020)** [41,42]	Ghana	PRONTO curriculum	Healthcare providers (nurses, midwives, doctors)	Maternal and newborn health; respectful maternity care	5 simulation scenarios covering obstetric and neonatal emergencies + 7 priority clinical topics (normal birth, newborn care, neonatal resuscitation, obstetric hemorrhage, preeclampsia/eclampsia, sepsis, preterm labor) + integrated RMC content emphasizing dignity, consent, autonomy, communication, privacy, and support for birth companions. Includes RMC-focused scenario (non-cooperative patient, squatting birth), structured debriefs, and guided self-reflection.	In-person, simulation-based training; includes role-play, interactive teamwork, and guided debriefs.
**Afulani 2023** [43]	Kenya	CPIPE	Maternity health care providers (nurses, midwives, doctors, clinical officers) and support staff working in antenatal, intrapartum, and postnatal units	Maternal health; respectful maternity care (RMC); provider wellbeing	Didactic and interactive training including the following content: Person-centered maternal care (PCMC); awareness of bias and strategies to mitigate it; managing difficult situations; integration of PCMC,	In-person training; uses vignettes, peer support groups, mentorship, and mindfulness exercises.
**Agu 2025** [44]	Nigeria	Action Learning Approach	Healthcare workers and managers	Adolescent & youth SRHR; gender-equitable services	Gender-transformative principles to address norms, power, and sexual rights in SRHR, using reflective diaries and participatory methods. It employs structured consensus-building approaches to identify and prioritize challenges in gender-equitable care, followed by action planning and follow-up meetings to support implementation and reflective learning	In-person sessions with WhatsApp follow-up; includes reflective diary writing and consensus-building exercises.
**Aktas 2021** [45]	Turkey	Empathy Training Program (ETP)	Midwives	Maternal health; respectful maternity care; empathy	Covers empathic listening, empathic reaction skills, and cognitive/affective dimensions of empathy.	In-person training; includes creative drama, psychodrama techniques, and role-playing.
**Antonakou 2025** [46]	Greece	Structured Educational Intervention	Midwives	Reproductive care; LGBTQ+ health	Covers gender equality, reproductive health rights, and inclusive care for LGBTQ+ individuals.	In-person training; includes case-based discussions and group dialogue.
**Asefa 2020** [47]	Ethiopia	RMC training manual	Midwives, doctors, nurses, health officers	Maternal health; respectful maternity care	Covers maternal health overview, human rights, RMC standards, professional ethics, and psychosocial components.	In-person (3-day) with on-site follow-up; includes participatory pedagogy, role-play, and video discussions.
**Avan 2023** [48]	Pakistan	S-RMC training	Maternity care providers (clinical/nonclinical); Nonclinical staff (traditional birth attendants/aayas (attendants), sweepers, security guards, and ward boys	Maternal health; respectful maternity care	Leadership and team-based maternity care; supportive and dignified maternity care; clarification of professional values to practice S-RMC; ethical, rights-based, and woman-centered care; psychosocial support; operationalization of S-RMC strategy	In-person training; includes reflection on patient-centered care and team coordination.
**Bacchus 2010** [49]	United Kingdom	Domestic Violence Training Programme	Health professionals (midwives, doctors, nurses)	Maternal/sexual health; Gender-based violence	Curriculum/training program introducing domestic violence clinical guidelines; one-day domestic violence training; training packs with information and resources available in clinics	In-person training; includes role-play, DVD demonstrations, and case discussions.
**Bagherzadeh 2025** [50]	Iran	Consultation-based ethical training	Midwives	Maternal health; respectful maternity care	Three-session curriculum covering seven dimensions of the Maternal Rights Charter … Ethical dilemmas discussed using moral case deliberation.	In-person group consultation; participants examine values/norms to propose solutions.
**Beckingham 2022** [51]	India	Midwifery training curriculum	Qualified nurses (GNM)	Maternal health; respectful maternity care	ICM competencies; respectful maternity care; women’s rights/choice; physiological labor and birth; antenatal, intrapartum, postpartum care; reflective practice; reducing unnecessary interventions.	Blended training
**BERGEN 2025** [52]	United States	Trauma-Informed Care (TIC) guide	Nursing students (Undergraduate)	Maternal health; sexual assault care	Trauma-Informed Care (TIC) training guide/safety, trust, transparency, peer support, collaboration, empowerment, cultural/historical/gender issues; communication skills with SA survivors; scenarios for adult female, teen, and male survivors; debriefing/reflection sessions	In-person training; includes role-play scenarios, debriefing, and reflection.
**Bermele 2018** [53]	United States	IPV education module	Registered nurses (L&D)	Maternal health; GBV/IPV	Nurses were educated on IPV, methods for screening… and a brief intervention.”; “The session includes information on the cycle of violence, orders of protection… local resources…”; “videotaped vignette…pregnant woman with abusive partner present…	Blended (Online module and live session; includes role-playing and video vignettes).
**Blair 2013** [54]	United States	Female Sexual Health Workshop	Medical/Mental health professionals	Sexual health	Topics: prevalence, classification, and diagnosis of female sexual dysfunction; impact on quality of life; barriers to treatment; overview of healthy sexual functioning; evidence-based treatment options (biological and psychosocial); referral pathways; PLISSIT model; bibliotherapy; normalization of sexual concerns; addressing clinician discomfort	In-person workshop; includes role-play dyads and peer feedback.
**Block 2022** [55]	United States	ECHO LGBTQ training	Oncology allied health professionals	Reproductive health; LGBTQ-inclusive care	LGBTQ cultural humility; SOGI data collection; Health disparities & barriers; Inclusive communication; Sexual + reproductive health for LGBTQ AYAs; Fertility preservation conversations; Intersectionality & Critical Race Theory lens; Case-based practice on introductions, assumptions, dating, sex, inpatient care	Online (asynchronous); includes interactive case-based modules and action plans.
**Boga 2025** [56]	Kenya	Emotional competence course	Nurses and nutritionists	Maternal and newborn health; RMC	Focus on: emotional triggers, self-awareness, communication behaviors, relational dynamics, respectful interactions, managing distress, strengthening team and parent relationships“…strengthening participants’ awareness of their own, their colleagues’, and their patients’ emotional triggers and responses.”; “enhancing relationships among staff and with parents…”	Blended learning; includes on-the-job reflection, workshops, and role-modeling.
**Bourret 2025** [57]	Democratic Republic of Congo (DRC)	CAC curriculum / IPAS training	Midwives, physicians, nurses	Abortion care; contraceptive counselling	Comprehensive abortion care (information, management, contraceptive counselling, PAC); Manual Vacuum Aspiration for incomplete abortion: Woman-centered care: Psycho-social support: CAC legislation and scope of practice: Community health navigation roles	Primarily in-person; includes clinical skills training and curriculum education.
**Brienza 2005** [58]	United States	IPV Residency Training	Internal Medicine residents	Gender-based violence (IPV)	IPV definitions, prevalence, risk factors; How to screen, diagnose, intervene; Survivor narratives (video + shelter experience); Barriers to disclosure; Resource navigation and community resources; Confidential documentation; Effects of IPV on health, work, children, home	In-person seminar; includes shelter-based training, role-play, and video.
**Brook 2001** [59]	Australia	Midwifery Curriculum (Feminist)	Nurses (midwifery students)	Maternal health; woman-centered care	Women’s stories and lived experiences; Childbirth education program content & process; Support groups (breastfeeding, postnatal, PND, homebirth); Interviewing women about physical, social, emotional impact of childbearing; Following a woman through pregnancy/labour/postpartum Reflection-on-action, feminist philosophy, praxis; Social, cultural, political influences on childbearing; Relationship-building with women.	In-person/Community-based; includes reflective tutorials, learning logs, and essays.
**Clerke 2023** [60]	Australia	Shared Decision Making (SDM)	Midwives and obstetricians	Maternal health (birth timing)	Shared decision-making using the Three-Talk Model, with role-play scenarios tailored to clinical guidelines for planned birth. It strengthens communication skills, rapport-building, and techniques to elicit women’s preferences and support informed, woman-centered decisions regarding birth options.	Blended online/workshops; includes simulation-based roleplay.
**Dagli 2025** [61]	Not reported	RMC Jigsaw training	Midwifery students	Maternal Health; RMC	Covers RMC concepts, equal care, dignity, confidentiality, and evidence-based practices.	In-person; uses Jigsaw Technique (cooperative learning).
**deDiego-Cordero 2022** [62]	Spain	Roma health intervention	Nursing students (1st year)	Gender and health; cultural competence	Covers Roma culture history, impact on health, stigma, and gender violence.	Online intervention.
**Dhakal 2022 & 2023** [63,64]	Nepal	RMC Online Education Module	Nursing students (BSN)	respectful maternity care	Principles and domains of RMC, and drivers of disrespect and abuse, and practical strategies to promote respectful, rights-based maternity care.	Online synchronous learning; includes reflective writing guide (Bass Model).
**Doherty 2025** [65]	South Africa	RMC Toolkit / PLA	Health professionals (Maternity)	Maternal and newborn health; PCMC	Covers Participatory Learning & Action, team roles, RMC concepts, and advocacy.	In-person team learning; includes role plays, vignettes, and mentorship.
**Dullius 2023** [66]	Brazil	Over the Rainbow (m-Health)	Health professionals	LGBT+ population health	Multi-module curriculum addressing sexuality, equity, cultural competence, public policy, and mental health to challenge provider assumptions while promoting inclusive, rights-based care for LGBT+ populations.	Online (mobile application).
**DuMont 2020 [**67]	Canada	Trans-affirming care curriculum	Forensic nurses (SANE)	GBV / Sexual assault; gender-affirming care	Competency-based training focused on assessment, clinical and forensic care, communication, and appropriate gender-affirming referral pathways for survivors, emphasizing respectful and appropriate service delivery	In-person classroom; includes interactive case-based discussions.
**Dzomeku 2021** [68]	Ghana	Respectful Maternity Care Modules	Midwives	Maternity care; RMC	Respectful childbirth practices emphasizing women’s rights, ethical care, effective communication and consent, focused antenatal care, and supportive birthing positions.	In-person training; includes role-play, discussion, and case studies.
**Edie 2020** [69]	United States	Gender and Women’s Health course	Nursing students	Women’s health; reproductive justice	Women’s Health Agenda; medicalization; reproductive justice; politics of abortion; violence against women; gender norms; societal, cultural, political influences	Online course; includes reflection, forum discussion, and assignments.
**El-Sheikh 2025** [70]	Egypt	Respectful Maternal Care Curriculum	Nursing students (3rd year)	Maternal health	Covers RMC definitions, human rights, types of disrespect, and barriers.	In-person; includes role-play and interactive discussions.
**Geddes 2017** [71]	Malawi	RMC (PANEL approach)	Midwives, managers	Maternal health	Human rights–based maternal care training using the PANEL framework and case studies to examine accountability, equity, and empowerment while strengthening respectful maternity care practices.	In-person; includes case study discussions and role-play.
**Gunn 2006** [72]	Australia	ANEW educational intervention	Midwives, medical practitioners	Antenatal care; psychosocial support	Workshop-based training using role-play and practical tools to strengthen woman-centered communication and provider responses to psychosocial and violence-related issues in maternity care.	In-person workshops; includes role-play and fishbowl technique.
**Henrich 2024** [73]	United States	Women’s Health / Gender-Affirming Care	Internal Medicine residents	Reproductive health; gender-affirming care	Women’s health and gender-affirming care, integrating equity, inclusive language, trauma-informed approaches, and rights-based SRHR across the life course.	Blended learning; includes case-based learning and skills practicums.
**Herring 2025** [74]	United States	Pediatric SGM E-learning module	Pediatric nurses	SGM health in pediatrics	Training focused on improving respectful, inclusive healthcare for pediatric sexual and gender minority patients, with emphasis on mental health and gender identity–affirming care.	Blended online/in-person; includes interactive case-based learning.
**Hetherington 2023** [75]	Tanzania	TAMANI curriculum	Healthcare providers, community health workers	Maternal and newborn health; adolescent-friendly services	Training and mentoring in basic and comprehensive EmONC, Respectful Maternity Care (RMC), comprehensive SRH services, adolescent-friendly services, community health, supportive supervision, maternal death reviews, HMIS strengthening, Social Analysis and Action (gender dialogue), quality improvement, and inclusive health planning.	In-person, multicomponent capacity-building programme combining training, mentoring, supportive supervision, quality supervision visits, participatory Social Analysis and Action (SAA) dialogues, community engagement, and health systems strengthening
**Hill 2024** [76]	United States	RMC Guideline Implementation	Registered nurses	Maternal health; RMC	AWHONN RMC framework, emphasizing shared decision-making, informed consent, autonomy, dignity, accountability, and advocacy to support respectful, patient-centered maternity care.	Blended (webinar, journal club); includes huddles.
**IdaMacleod 2024** [77]	South Africa	Abortion Counselling Certificate	Nurses, abortion providers	Abortion counselling	Person-centered, evidence-based abortion counseling that integrates reproductive justice, informed consent, and confidentiality while addressing cultural norms, religion, and gendered power relations in complex care scenarios.	In-person training; includes role-plays (CARM) and reflective journals.
**Jack 2019** [78]	United States	Nurse-Family Partnership IPV	Registered nurses	GBV / IPV services	Skills-based IPV intervention focused on empathic assessment, risk evaluation, safety planning, and referral, with education on health effects and strategies to enhance survivor self-efficacy and system navigation.	In-person training; includes application exercises and reflective supervision.
**Jungels 2023** [79]	United States	Prenatal Genetic Testing Module	Genetic counselors, doctors, nurses	Genetic counseling	Educational module combining patient-centered care and shared decision-making with vignettes and implicit bias assessment to examine attitudes toward people with intellectual disabilities and improve equitable care.	Web-based module; includes clinical vignettes and IATs.
**Kataoka 2004** [80]	Japan	Sexual Assault Nursing Program	Nurses	GBV / Sexual assault response	Targets nurses’ attitudes toward sexual assault survivors, explicitly addressing rape myths while strengthening knowledge and skills for respectful, survivor-centered care.	In-person structured program.
**Kim 2002** [81]	South Africa	Gender Violence Training Module	Primary health care nurses	GBV and reproductive health	Examine nurses’ attitudes and roles in responding to gender-based violence, addressing barriers to action while building practical skills for detection, referral, and community education.	In-person; includes role reflection and scenario discussions.
**Kostu 2022** [82]	Turkey	IPV Training Program	Nurses and midwives	GBV / IPV services	Cover gender equality and violence-prevention content with legal, communication, and clinical skills to strengthen nurses’ and midwives’ roles in delivering rights-based responses to IPV	In-person; includes movie screening, role play, and discussion.
**Kramer 2025a** [83]	United States	Implicit Bias Training	Maternal healthcare professional	Maternal Health/Perinatal care	Covers implicit bias, structural racism, racial disparities, mistreatment, equity vs. equality	Online / Asynchronous training
**Kramer 2025b** [84]	United States	Perinatal SUD Bias Reduction	Maternal healthcare professionals	Maternal Health/Substance Use Disorder (SUD)	Address stigma, bias, and punitive responses to substance use in pregnancy, integrating lived-experience vignettes and supportive care strategies	Online / Asynchronous training
**Kumari 2024** [85]	India	Respectful Abortion Care (RAC)	Obstetricians and Gynaecologists	Respectful Abortion Care	Covers modular abortion care training integrating legal literacy, values clarification, stigma reduction, and quality-of-care principles with up-to-date clinical guidance	Online training; uses WHO value clarification exercises.
**Macker 2023** [86]	United States	Trans* Survivor Care Course	Sexual Assault Nurse Examiners	Gender affirming care for SA survivors	Structural-competency-informed training on caring for trans* survivors, addressing intersecting identities, health inequities, and violence while strengthening respectful, gender-affirming clinical and forensic practice	Online course; includes structural competency framework.
**Mann 2024** [87]	United States	Intro to Transgender Healthcare	Nurse Residents	Gender affirming SRH care	Covers education on transgender health and cultural humility with case-based discussion; transphobia and promote respectful, trauma-informed reproductive care.	Blended virtual/in-person; includes case study and role-play.
**Manu 2023** [88]	Bangladesh/Ghana/Tanzania	MNH Quality of Care standards	Health workers (doctors, nurses)	Maternal/newborn care; RMC	Covers compassionate, person-centered care, emphasizing effective communication, informed consent, shared decision-making, client feedback, and creation of a safe, dignified care environment.	In-person training structured within QI initiatives.
**Mayfield 2017** [89]	United States	LGBTQ Health Curriculum	Medical students	Sexual health; LGBTQ health	Covers sexual history-taking skills with LGBTQ-inclusive communication and implicit bias reflection to improve equitable, respectful sexual health care.	Blended; includes standardized patients, role-play, and bias activity.
**McGrew 2025 [**90]	United States	Generative AI Telehealth Simulation	Midwifery students	Maternal health; culturally responsive care	Case-based training focused on delivering person-centered, culturally responsive telehealth care for underserved populations.	Online simulation (AI avatar); includes structured debrief.
**McLachlan 2011** [91]	Australia	PinC ANEW education programme	Midwives	Postnatal care; psychosocial support	Covers Postnatal psychosocial issues (depression, IPV, isolation, past abuse, substance use, anxiety) and communication skills	In-person workshops; includes role-play and video exercises.
**Mena-Tudela 2020** [92]	Spain	Obstetric Violence Intervention	Nursing/Medicine students	Maternal health; obstetric violence	Covers Obstetric violence & RMC; theatrical portrayal; legal aspects; professional perspectives; mothers’ lived experiences	In-person seminar; includes theatrical performance and round tables.
**Mengistu 2021** [93]	Ethiopia	RMC Testimonial Video Intervention	Maternity providers	Maternal/newborn health; RMC	Covers reflection; mistreatment prevalence; empathic communication; QI problem-solving (PDSA cycles)	In-person includes testimonial videos and participatory reflection.
**Miller 2017** [94]	United States	ARCHES Intervention	Clinic personnel	Family planning; IPV	ARCHES trauma-informed, survivor-centered SRHR framework (education, assessment, harm-reduction counseling, warm referral, scripts, educational cards)	In-person training; includes scripts and workflow exercises.
**Mishkin 2024** [95]	United States	Breaking Through Bias	Maternal health clinicians	Maternal health; racial equity	Covers Implicit bias & structural racism in maternity care; mitigation strategies; culture of equity	Blended (live/self-paced); includes interactive group conversations.
**Mosley 2020** [96]	Multi-country	Abortion Stigma Workshop	nurses, midwives, physicians, counselors, laboratory and ultrasound technicians, and reception staff involved directly or indirectly in abortion or post-abortion care	Abortion care; stigma reduction	Reflective intervention on stigma, identity, community context, and emotional challenges to support values clarification and professional resilience.	In-person workshop; includes storytelling and arts-based methods.
**Narino 2025** [97]	Brazil	CAM Anti-Racist Leadership	Maternity leaders	Maternal health; equity	Covers anti-racism, structural racism, and race-conscious care.	In-person/virtual; includes group discussions and case reflection (Blended)
**Nathan 2020** [98]	United States	SAFERR Curriculum	Advanced practice nurses	Sexual assault care	Trauma-informed care for sexual violence & trafficking survivors (TIC, consent, forensic skills, simulations, trans-inclusive care)	In-person (lectures/simulation; includes standardized patients).
**Ndwiga 2017** [99]	Kenya	Heshima project (VCAT)	Healthcare workers	Maternal health	Covers Values, attitudes & belief transformation; RMC; childbirth rights; consent, privacy, teamwork	In-person workshops; includes VCAT exercises.
**Nigenda 2025** [100]	Mexico	Birthing Centered Model Curriculum	Obstetric Nurse interns	Maternal Health; RMC	Covers social medicine and values for vulnerable populations.	In-person induction and supervised care.
**Odimba 2021** [101]	DRC	Collaborative Learning Guide	Healthcare workers	Adolescent SRH	Covers rights, ethics, and values alongside clinical content, communication skills, confidentiality, and empathetic care	In-person; includes case studies and group reflection.
**Ogolla 2024** [102]	Kenya	CPIPE	Maternity health care providers (nurses, midwives, doctors, clinical officers) and support staff working in antenatal, intrapartum, and postnatal units	Maternal health; PCMC	Covers PCMC, bias awareness, stress and burnout management, mindfulness, teamwork, and emergency obstetric simulations.	In-person workshop/simulation; includes peer support groups.
**Ontiri 2025** [103]	Nigeria	DISC empathy-based training	Family planning providers	Family Planning	Covers empathetic counseling, values clarification, person-centered care, self-injection, systems strengthening	Blended; includes role-play and practicum.
**Ouédraogo 2014** [104]	Burkina Faso	RMC Training	Obstetricians, midwives	Maternal Health; RMC	Covers respectful care and interpersonal skills.	In-person; includes role play and case studies.
**Paluzzi 2000** [105]	United States	Domestic Violence Education	Nurse-Midwives	GBV / Domestic Violence	Covers DV screening, referral, and cultural competence.	Blended (Workshop/Video); includes distance learning.
**Park 2013** [106]	South Korea	Cultural competence program	Maternity nurses	Maternal health	Multicultural maternity care curriculum (3-D Puzzle Model; transcultural nursing; cases; negotiation & reflection)	In-person case-based small group learning.
**Ray 2023** [107]	Zimbabwe	RMC educational package	Midwives	Maternal Health; RMC	Covers 12-week transformative learning + RMC Charter (rights, autonomy, power, consent, dignity, self-care, mistreatment)	In-person workshop; includes reflective writing journals.
**Rege 2019** [108]	India	Gender-integrated curriculum	Medical students	Gynecology / Reproductive Health	Covers social construction of gender, obstetric violence, and GBV.	In-person lectures; includes role play and debates.
**Rishard 2024** [109]	Sri Lanka	RMC workshop (Buddhist)	Nurses, midwives, doctors	Maternal Health; RMC	Covers RMC linked to spiritual principles (Metta, Karuna).	In-person interactive workshop.
**Roemer 2024** [110]	Kenya and Tanzania	VCAT-based RMC Toolkit	Maternity staff	Maternal and Neonatal Care	Covers bodily autonomy, power/gender dynamics, and obstetric violence.	Blended (Online/In-person); includes VCAT exercises.
**Rosser 2025** [111]	Tanzania	Comprehensive Sexual Health	Medical/Nursing students	Sexual health	Covers sexual health, key populations, and sexual violence.	In-person lectures/simulation; includes role-play.
**Rubyan 2025** [112]	United States	ArtSpective for PSU	Perinatal nurses	Maternal health (Substance Use)	Covers perspective taking and stigma/implicit bias.	Online platform; includes perspective-taking exercises.
**Sari 2025** [113]	Indonesia	Health ethics module	Midwives	Maternal health	Covers ethical decision-making and empathetic counseling.	In-person mentoring and supervised reading.
**Saxell 2009** [114]	Canada	Maternity Care Club	Medical/Nursing students	Maternal and newborn health	Covers labour support, substance use, and care for marginalized women.	In-person (lectures/simulation; includes service-learning).
**Schoening 2004** [115]	United States	DV Coordinating Council	Inpatient nurses	GBV / IPV	Covers IPV dynamics, screening, and interventions.	In-person video/lecture; includes interactive discussion.
**Seybold 2014** [116]	United States	SAMHSA Model Foundations	Healthcare practitioners	Maternal health (Substance Use)	Covers addiction, compassion/bias, and professional readiness.	In-person conference; includes role-play and panel.
**Shaikh 2006** [117]	Pakistan	Health Workers for Change	Health workers	Reproductive health	Covers gender/women’s status, motivations, and obstacles.	In-person participatory workshops; includes role-play.
**Sharma 2022** [118]	India	RMC teaching programme	Staff nurses	Maternal health	Covers RMC components, domains, and policies.	In-person training.
**Singh 2025** [119]	India	RMC Curriculum (USAID)	Healthcare providers	Maternal health	Covers rights of mothers, dignity, and respectful care.	In-person training; includes practical sessions.
**SisCelik 2019** [120]	Turkey	Violence Against Women Course	Student midwives/nurses	Gender-based violence	Covers social & gender equality, violence types, and legal aspects.	In-person classroom lectures.
**Skalisky 2025** [121]	United States	Midwifery Academic Course	Midwifery students	Maternal/Reproductive health	Covers implicit bias, structural racism, and power dynamics.	Blended; includes self-reflection and advocacy project.
**Smith 2013** [122]	Multi-country	CCSAS	Healthcare providers	Sexual assault care	Covers beliefs affecting survivors, rights, and compassionate care.	In-person; includes video re-enactment and role plays.
**Smith 2018** [123]	Australia	DV Screening Workshop	Midwifery students	Maternal health; DV screening	Covers screening, responding safely, and self-care.	In-person; includes observed role-play.
**Smith 2025** [124]	United States	Respectful Maternal Care Manual	Maternity providers	Maternal health	Covers implicit bias, RMC principles, and autonomy.	In-person; includes video scenarios and team discussions.
**Soomar 2025** [125]	Pakistan	Gender Identity Curriculum	Nursing students	Gender diversity; inclusive care	Covers gender identity, stigma, and human rights.	In-person & online (blended); includes case-based discussions.
**StaceyMitchell 2023** [126]	United States	Virtual Reality SANE simulation	Registered Nurses (SANE)	Sexual Assault Care	Covers exam skills, trauma-informed care, and evidence.	Simulation-based with VR modules (Online).
**Steen 2003** [127]	United Kingdom	In-service DV Training	Midwives	Domestic violence	Covers personal accounts of DV and professional competence.	In-person; includes reflective practice.
**Stevens 2002** [128]	Not reported	Program guide (GBV)	Health care providers	Gender-based violence	Covers GBV assessment, sexual abuse, and psychosocial effects.	Program guide (adaptable).
**Suarez 2025** [129]	United States	Sexual Health Curriculum	Internal medicine residents	Gender/sexual diverse patients	Covers sexual health, PrEP, and gender-affirming hormone therapy.	In-person; includes case-based learning.
**Sung 2016** [130]	Taiwan	Sexual Health Training	Registered nurses	Sexual healthcare	Covers sexual orientation, gender identity, and harassment.	In-person; includes case studies and role-play.
**Swordy 2020** [131]	United Kingdom	Footprints of Birth	Mixed health professions	Maternal care; RMC	Documentary of women’s video-selfies followed by reflection.	In-person workshop; includes film and reflective practice.
**Tajvar 2025** [132]	Iran	RMC Educational Intervention	Midwives	Maternal health; RMC	Covers RMC dimensions, mistreatment, and autonomy.	In-person; includes scenario creation and film screening.
**Tumwine 2023** [133]	Multi-country (LMIC)	ITP in SRHR	Healthcare practitioners	Broad SRHR	Covers youth sexual health, HIV, and sexual identity.	In-person; includes collaborative learning and value exercises.
**Ufomata 2020** [134]	United States	LGBT Health Curriculum	Internal medicine residents	LGBT health	Covers LGBT issues, cultural competencies, and violence.	In-person; includes case-based guided questions.
**UnalToprak 2021** [135]	Turkey	SRH Education Curriculum	Nursing students	SRH education	Covers gender, sexuality concepts, rights, and violence.	In-person classroom; includes role-playing.
**Vakily 2017** [136]	Iran	DV Educational Module	Midwives	GBV services	Covers DV prevalence, ideas of Islam, and screening.	In-person group sessions and self-study (Blended)
**Wang 2023** [137]	Taiwan	LGBT Cultural Competence	Student nurses	LGBT health	Covers LGBT concepts, culturally competent care, and simulation.	Online/Simulation-based.
**Webber 2018 & 2020** [138,139]	Tanzania	Health Workers for Change	Nurses	Reproductive health	Covers Health Workers for Change curriculum.	In-person; includes participatory exercises and role plays.
**White 2020** [140]	United States	Graduate Sexual Health Course	Graduate nursing students	Sexual/reproductive health	Covers disparities, LGBTQ+ care, and history-taking.	Blended (In-person seminar; includes role-play, online discussion
**White 2022** [141]	United States	Substance Abuse Education	Staff nurses	Substance use in pregnancy	Covers substance abuse effects and stigma barriers.	Online PowerPoint presentation.
**Wilson-Mitchell 2018** [142]	Tanzania	RMC Workshop	Midwives	Maternal health; RMC	Covers 7 domains of RMC, human rights, and equity.	In-person workshop; includes role plays.
**Yilmaz 2018** [143]	Turkey	Gender Equality Course	Midwifery students	GBV within SRHR	Covers gender roles, inequality, and domestic violence.	In-person; includes case reports and dialogue.

### Characteristics of the 25 toolkits/curricula/manuals

The majority of the toolkits/manuals targeted healthcare providers (92% (23/25)). Nearly half also included trainers, managers, or policymakers (44% (11/25)), reflecting a focus on capacity building and system-level implementation. A smaller proportion targeted community or non-clinical personnel (12% (3/25)) and students or trainees (8% (2/25)). Because some toolkits targeted multiple participant groups, these categories are not mutually exclusive.

Toolkits/manuals addressed a broad range of SRHR content domains. The most frequently represented were **gender norms, equity, and rights (15/25, 60%)** and **abortion care and stigma (11/25, 44%). Gender-based violence (6/25, 24%)** and **adolescent and youth** SRHR **(5/25, 20%)** were also commonly addressed, followed by **family planning (4/25, 16%).** Fewer toolkits focused on LGBTQ+ **inclusive care (2/25, 8%), respectful maternity care (2/25, 8%)**, and **sexual health, cultural competence, and provider wellbeing (2/25, 8%). Female genital mutilation (1/25, 4%) a**nd **disability inclusion (1/25, 4%)** were rarely addressed. Because toolkits frequently covered multiple thematic areas, content domains were not mutually exclusive and percentages exceed 100%.

Pedagogically, experiential learning (100%) and reflective or values-based approaches (96%) were nearly universal. These approaches frequently incorporated role-play, case studies, simulation, value clarification, and facilitated reflection to strengthen communication skills, clinical decision-making, and person-centered care.

Nearly all toolkits emphasized equitable or rights-based care (24/25, 96%), while many also incorporated gender-transformative (20/25, 80%) and system-level (23/25, 92%) elements. Most toolkits/manuals included components targeting both individual- and structural-level influence. Individual-level components (20/25, 80%) focused on strengthening provider knowledge, attitudes, communication, and clinical skills, whereas structural-level components (23/25, 92%) addressed policies, organizational practices, service delivery models, and broader health system strengthening. Because these levels of influence were not mutually exclusive, many toolkits incorporated strategies across more than one level.

### Characteristics of the 100 interventions

#### Target populations.

All interventions targeted current or future healthcare workers involved in SRHR service delivery, including practicing providers, trainees, residents, and students (Table 2). The majority focused on frontline providers, including physicians, nurses, and midwives (64/100, 64%). A smaller proportion targeted pre-service trainees exclusively (17/100, 17%), including medical, nursing, and midwifery students. Several interventions targeted mixed participant groups, including both providers and trainees (11/100, 11%). In addition, a subset of interventions included multidisciplinary or non-clinical participants, such as community health workers, support staff, managers, or policymakers (8/100, 8%).

#### Delivery modalities and reinforcement strategies.

Most interventions were delivered in person (66/100, 66%), with smaller proportions delivered online (15/100, 15%) or through blended formats combining in-person and online approaches (18/100, 18%), or using **adaptable delivery formats (1/100, 1%) (**Table 2**).** Simulation-based training was used across delivery modalities, including in-person simulation, standardized patient encounters, and emerging digital formats such as virtual or AI-based simulations. Reinforcement strategies, including mentorship, follow-up sessions, peer support groups, and quality improvement (QI) cycles, were incorporated in over half of interventions (61/100, 61%). These approaches were used to facilitate integration of training into clinical practice. The remaining interventions either did not report structured reinforcement strategies or were delivered as standalone training interventions (Table 2).

#### Content domains.

The 100 interventions covered a wide range of SRHR topics (Table 2). The most common domain was maternal and newborn health, particularly respectful maternity care (RMC) and person-centered maternity care (PCMC), representing 52/100 (52%) of interventions. These interventions emphasized dignity, informed consent, communication, and respectful treatment during childbirth. Interventions addressing gender-based violence (GBV) and intimate partner violence (IPV) accounted for 25/100 (25%) of interventions and focused on screening, survivor-centered care, and referral pathways. Gender norms, equity, and rights were also a prominent focus (31/100, 31%), often integrated across training content to address provider bias and promote equitable care. A substantial proportion of interventions addressed LGBTQ+ inclusive care (22/100, 22%)**,** reflecting efforts to improve culturally competent and inclusive SRHR service delivery. Smaller proportions of interventions focused on sexual health and counseling (10/100, 10%)**,** cultural competence and marginalized populations (10/100, 10%), and abortion care and stigma reduction (9/100, 9%), often incorporating values clarification and counseling approaches.

Additional domains, including family planning (6/100, 6%)**,** substance use in pregnancy (6/100, 6%)**,** provider wellbeing (7/100, 7%)**,** and adolescent SRHR (3/100, 3%)**,** were less frequently represented and were often integrated within broader training approaches. Several interventions also addressed broader issues of equity, stigma, and bias, including implicit bias, structural racism, and discrimination in SRHR care, although these were typically embedded across domains rather than presented as standalone training areas. Content domains were not mutually exclusive; **thus, individual interventions could be assigned to more than one domain; therefore, percentages do not sum to 100%.**

#### Pedagogical approaches.

Across the interventions (Table 3), didactic instruction (98/100, 98%), reflective and value-based approaches (80/100, 80%), and experiential learning approaches (90/100, 90%) were commonly used. Reflective approaches typically involved facilitated discussion, journaling, and value clarification activities, whereas experiential approaches involved simulation, role-play, case-based learning, and scenario-based exercises. Didactic instruction was rarely used as a standalone strategy and was typically combined with experiential and reflective methods. Together, these approaches supported both competency development and critical reflection on provider attitudes, values, and biases.

**Table 3:** Distribution of training characteristics across toolkits/manuals and interventions.

Groupings	Toolkits/Manuals (N = 25 (%*))	Intervention components (N = 100 (%*))	All records (N = 125 (%*))
**Pedagogical approaches and intervention characteristics**			
Experiential learning present	25 (100%)	90 (90%)	115 (92%)
Reflective/values-based approaches	24(96%)	80 (80%)	104 (83.2%)
Didactic instruction present	25 (100%)	98 (98%)	123 (98.4%)
Digital/online component	2 (8%)	37 (37%)	39 (31.2%)
Reinforcement/support strategies	20 (80%)	61 (61%)	81(64.8%)
Equitable/rights-based care	24 (96%)	99 (99%)	123 (98.4%)
Gender-transformative elements	20 (80%)	66 (66%)	86 (68.8%)
System-level elements	23 (92%)	70 (70%)	93 (74.4%)
**Level of influence**			
Individual-level focus	20 (80%)	100 (100%)	120(96%)
Interpersonal-level focus	1 (4%)	86 (86%)	87 (69.6%)
Structural-level focus	23(92%)	69 (69%)	92 (73.6%)

* *Percentages for pedagogical approaches, intervention characteristics, content domains, and levels of influence were calculated using the total number of toolkits/manuals (n = 25) or intervention components (n = 100) as the denominator. These categories were not mutually exclusive; therefore, a single toolkit/manual or intervention component could be assigned to more than one category, and percentages do not sum to 100%.*

#### Transformative focus and level of change.

Across interventions, there was a strong emphasis on equitable, respectful, and rights-based care. Many interventions aimed to improve communication, enhance empathy, and reduce stigma in clinical interactions. Gender-transformative elements were present in (66/100, 66%) interventions. System-level elements were incorporated in 70/100 (70%) of interventions, reflecting institutional change, policy engagement, or organizational accountability mechanisms. These were often embedded within broader quality improvement or leadership initiatives.

Across the included intervention studies, **all interventions (100%)** included **individual-level components**, targeting healthcare providers’ knowledge, attitudes, values, confidence, competencies, or clinical skills (Table 3). **Interpersonal-level components** were identified in **86/100 (86%)** of interventions and focused on strengthening provider–patient communication, respectful and person-centered care, empathy, shared decision-making, and therapeutic relationships. **Structural-level components** were identified in **69/100 (69%)** of interventions and addressed broader organizational or health system factors, including institutional policies, clinical guidelines, curricula, leadership, quality improvement initiatives, implementation strategies, and health system processes. Because levels of influence were not mutually exclusive, many interventions addressed multiple levels simultaneously.

## Discussion

This scoping review identified a diverse body of tools and educational interventions aimed at strengthening healthcare workers’ competencies and addressing gender bias in sexual and reproductive health and rights (SRHR) service delivery. Across the included materials, 25 training tools and 100 intervention approaches were identified, spanning multiple SRHR domains, targeting a wide range of healthcare cadres, and geographic contexts. Overall, the findings highlight the breadth and characteristics of the available literature, although the evidence base remains uneven across regions, SRHR topics, and types of training approaches.

### Pedagogical approaches and learning strategies

A consistent pattern emerged across both tools/manuals and evaluated interventions: most approaches emphasized respectful, person-centered, and rights-based care, frequently combining multiple pedagogical strategies rather than relying on a single training method.

- Didactic approaches were commonly used to provide foundational knowledge on SRHR, gender, stigma, and rights-based care.
- Reflective and value-based approaches, including values clarification and attitude transformation (VCAT), facilitated discussion, and guided reflection, were widely employed to support providers in examining their beliefs, attitudes, and biases.
- Experiential learning approaches, such as simulation, role-play, and case-based learning were frequently used to strengthen communication, counselling, and clinical skills.

Across both toolkits/manuals and intervention studies, didactic instruction was almost universal and was commonly integrated with experiential and reflective learning approaches. This suggests that intervention developers rarely relied on knowledge transmission alone, instead combining cognitive, behavioral, and reflective learning strategies to address provider bias and promote equitable care. These findings align with adult learning principles and reflect efforts to address complex, socially embedded issues such as stigma and discrimination in SRHR care. This pattern is consistent with evidence that effective professional education mobilizes multiple, integrated learning methods rather than relying on a single approach and with findings from participatory approaches, which engage health workers in critical reflection and collective learning to improve attitudes and practices [144,145]. The reviewed interventions commonly combined these approaches, reflecting current educational practice for addressing complex SRHR competencies and provider bias.

Many interventions incorporated system-level components alongside educational, mentorship, quality-improvement, or implementation-support strategies, reflecting efforts to address organizational and health-system factors in addition to provider competencies. Similarly, although gender-transformative elements were identified in many interventions, the extent of their integration varied across studies.

### SRHR content areas and service delivery focus

A key thematic concentration was observed in maternal and newborn health services, particularly respectful maternity care (RMC) and person-centered maternity care, which accounted for over half of all interventions. These programs focused on improving dignity, communication, informed consent, and respectful treatment during childbirth, reflecting longstanding global concerns about mistreatment in facility-based maternity care and the recognition of respectful care as a fundamental human rights issue [146,147]. Despite this focus, relatively few interventions explicitly included non-clinical personnel working in maternity settings, such as ward attendants or support staff, who also influence patient experiences. This represents an important gap in efforts to promote respectful care across the full continuum of service delivery.

Beyond maternity care, interventions addressed a range of SRHR topics, including gender-based violence (GBV), intimate partner violence (IPV), sexual assault response, abortion care, sexual and reproductive health, and LGBTQ+ inclusive care. Training programs in these areas commonly aimed to strengthen providers’ capacity to deliver survivor-centered and inclusive services, often through communication skills development and stigma reduction strategies. This aligns with broader evidence highlighting the critical role of health systems in responding to violence against women, alongside the persistent challenges in integrating comprehensive responses, particularly in low- and middle-income settings [148–150]. It also reflects growing evidence that culturally competent and inclusive care for sexual and gender minorities is critical to addressing health disparities and improving access to SRHR services [151].

### Stigma reduction, bias awareness, and gender-transformative approaches

Across SRHR topics, many interventions incorporated stigma reduction and bias awareness, often through values clarification and reflective learning activities. These approaches were intended to support healthcare workers in examining personal beliefs, social norms, and implicit biases that may influence clinical decision-making. This finding is consistent with evidence that stigma and discrimination remain important barriers to equitable and respectful SRHR care, particularly for marginalized populations, and that reflective and participatory learning approaches have been associated with cultural humility and improve provider attitudes and practices.

The mapped interventions commonly emphasized provider knowledge, attitudes, communication, and clinical practice while also incorporating structural or system-level components in some cases. However, the review identified variability in the extent to which broader organizational, policy, and health system factors were integrated into intervention design. Evidence suggests that changes in provider attitudes may be difficult to sustain without reinforcement mechanisms such as mentorship, supervision, organizational support, and routine systemic integration [152,153].

Many tools/manuals and interventions incorporated gender-transformative elements, including critical reflection on gender roles, power relations, and structural inequalities. However, although equitable and rights-based principles were widely incorporated across interventions, the extent of integration of gender-transformative approaches varied. This review highlights a need for further research examining how gender-transformative approaches are implemented, reinforced, and sustained within health systems.

### Key patterns and remaining gaps

Taken together, three consistent patterns emerged. First, most interventions were multi-component, frequently combining experiential, reflective, and didactic learning approaches. Second, interventions commonly sought to strengthen provider knowledge, attitudes, communication, and clinical practice, while many also incorporated organizational or system-level components. Approximately seven in ten interventions incorporated system-level elements (71%) and structural-level components (69%), indicating that many interventions extended beyond individual provider training to include organizational, policy, leadership, quality-improvement, and implementation strategies. Third, while equitable and rights-based care was widely promoted, the extent to which structural and gender-transformative elements were integrated varied across interventions.

### Limitations

This review should be interpreted in light of several limitations. It focused on documented tools and intervention approaches and may not capture all training activities implemented in practice. Online tools may not have been identified because access required registration or membership in professional associations. The geographic distribution of interventions was uneven, with a concentration in North America, Asia, and Africa and fewer reported in other regions. **Variability in how gender, equity, rights-based, and gender-transformative approaches were defined, operationalized, and reported across sources reduced consistency in how interventions could be classified and compared.** The coding of domains involved a degree of interpretive judgement by the research team, though consistency was supported through predefined criteria and regular discussion among reviewers.

Despite these limitations, the review provides a comprehensive mapping of tools and intervention approaches aimed at strengthening healthcare workers’ competencies and addressing gender bias in SRHR care. **The use of a structured coding framework, source-level coding matrix, and predefined decision rules strengthens the transparency and reproducibility of the synthesis.**

### Implications for research, policy, and practice

Strengthening gender-responsive SRHR care will require moving beyond isolated training interventions towards integrated, system-level strategies that engage organizational culture, leadership, accountability mechanisms, and policy environments. Training should be tailored to different healthcare cadres and supported by multidisciplinary, team-based approaches. Furthermore, supportive supervision, enabling policies, and institutional accountability mechanisms are needed to sustain long-term changes in provider behavior and reinforce equitable and non-discriminatory practices.

Integrating gender-transformative approaches into existing training frameworks may represent an important opportunity to strengthen impact. Collaboration between governments, academic institutions, and implementing organizations may support the scale-up, adaptation, and contextualization of effective approaches across diverse settings.

## Conclusion

This scoping review identified a substantial body of training tools and interventions designed to strengthen healthcare workers’ competencies and promote respectful, rights-based SRHR care**. Reflective, experiential, and rights-based approaches were widely incorporated across both tools/manuals and interventions, demonstrating increasing attention to provider attitudes, communication, and person-centered care.**

However, although equitable and rights-based principles were widely incorporated across interventions, the extent to which gender-transformative approaches were integrated varied. Future research should examine how gender-transformative approaches can be more consistently integrated alongside individual-, interpersonal-, and structural-level strategies, reinforcement mechanisms, and organizational support to strengthen equitable SRHR service delivery.

## Supporting information

S1 Data S1 File. Search strategy. **S2 File.** Excluded studies. **S3 File.** Supplementary codebook. **S4A.** Source-level coding matrix (Tools/Manuals). **S4B.** Source-level coding matrix (Interventions). **S5.** PRISMA-ScR-checklist.
(ZIP)
