# Supplementary material for: Addressing gender bias in sexual and reproductive health care: A scoping review of training tools and interventions
Source: PLoS One. 2026 Oct 13;21(10):e0359704. doi: 10.1371/journal.pone.0359704 (PMC13660924; doi:10.1371/journal.pone.0359704)
Supplement: Supplementary file 1 [file pone.0359704.s001.zip › Supporting Information S1 - 5/S2 File Excluded studies.docx]

**S2 File: Excluded Studies**

| **Study ID/Manual/Toolkit** | **Reasons for exclusion** |
| --- | --- |
| Baldacchino, R., & Abou Abbas, D. (2024). Sexual and reproductive health and rights in the Middle East and North Africa: A toolkit for rights- based, inclusive, and crisis-responsive action. Asfari Institute for Civil Society and Citizenship, American University of Beirut. | Humanitarian policy and system guidance; not training for HCWs; no structured curriculum. |
| Butt, A. P., Valerio, K., & Davies, I. (2020). Social norms diagnostic tool: Sexual and reproductive health and rights & gender-based violence. Oxford: Oxfam. | Not a health worker training manual. It is explicitly designed for programme teams, community members, and facilitators as a diagnostic and formative research tool, |
| Delivered by women, led by men: A gender and equity analysis of the global health and social workforce. Geneva: World Health Organization; 2019 (Human Resources for Health Observer Series No. 24). Licence: CC BY-NC-SA 3.0 IGO. | A gender and equity analysis report, not a training manual. |
| EngenderHealth & Promundo. Engaging Boys and Men in Gender Transformation: The Group Education Manual. New York: The ACQUIRE Project/EngenderHealth; 2008. ISBN 978-1-885063-77-9. | Not targeted to healthcare workers delivering SRHR services |
| EngenderHealth & Promundo. Engaging Men in Gender Equality and Health: A Global Toolkit for Action – Tools. Washington, DC/Rio de Janeiro: EngenderHealth/Promundo; 2009. | A facilitator/training toolkit; primarily designed for program implementers, NGOs, and community-based interventions |
| EngenderHealth & United Nations Population Fund (UNFPA). (2017). Engaging men in sexual and reproductive health and rights, including family planning. New York: UNFPA. | An educational component intended to inform and guide programme design and implementation |
| EngenderHealth. 2020. Gender, Youth, and An Staff Training Manual. Washington, DC: EngenderHealth. | Participants is not healthcare worker even though it is a structured training manual, with reflective practices and a strong SRHR focus |
| EngenderHealth. Gender, Youth, and Social Inclusion (GYSI) Analysis Framework and Toolkit. EngenderHealth; year not specified. | An analysis and program design toolkit, not a training manual |
| Extending Service Delivery (ESD) Project. Healthy timing and spacing of pregnancy: A trainer’s reference guide (2nd ed.). Washington, DC: Pathfinder International; 2010. Supported by the U.S. Agency for International Development (USAID) | A trainer’s reference guide focused on technical family planning counseling and service delivery (HTSP) |
| Family planning and comprehensive abortion care toolkit for the primary health care workforce. Volume 1. Competencies. Geneva: World Health Organization; 2022 (Family planning and comprehensive abortion care toolkit for the primary health care workforce). | A policy/guidance/tool document, not a training curriculum or educational intervention |
| Family planning and comprehensive abortion care toolkit for the primary health care workforce. Volume 3. Dissemination, implementation, monitoring and evaluation (DIME). Geneva: World Health Organization; 2023 (Family planning and comprehensive abortion care toolkit for the primary health care workforce). | This volume provides implementation guidance, dissemination strategies, monitoring & evaluation frameworks, stakeholder engagement, and system-level adoption strategies |
| Family planning and comprehensive abortion care toolkit for the primary health care workforce. Volume 2. Programme and curriculum development guide. Geneva: World Health Organization; 2022 (Family planning and comprehensive abortion care toolkit for the primary health care workforce). Licence: CC BY-NC-SA 3.0 IGO. | A programme and curriculum development guide. “designed for those “preparing or revising formal education and training programmes and curricula” |
| ICM Essential Competencies for Midwifery Practice. The Hague: International Confederation of Midwives; 2024. Licence: CC BY-NC-SA 4.0. | A professional competency framework, not a training curriculum, manual, toolkit, or instructional program. |
| International Association of Providers of AIDS Care (IAPAC). (2015). Train-the-trainer manual: HIV clinical management. International Association of Providers of AIDS Care. Supported by a restricted educational grant from Gilead Sciences. | Purely biomedical HIV content |
| International Planned Parenthood Federation (IPPF). (2010). Men are changing: Case study evidence on work with men and boys to promote gender equality and positive masculinities. London: IPPF. | A research report and synthesis of case studies, not a training manual, curriculum, toolkit |
| International Planned Parenthood Federation (IPPF). (2017). Deliver and Enable: A Toolkit to Strengthen Comprehensive Sexuality Education. London: IPPF. | An advocacy and comprehensive sexuality education (CSE) toolkit aimed at educators and programme actors |
| Ipas. 2020. The full story: Advocating for comprehensive sexuality education that includes abortion. Chapel Hill: Ipas. | An advocacy toolkit |
| IPPF and UNFPA (2017). Global Sexual and Reproductive Health Service Package for Men and Adolescent Boys. London: IPPF and New York City: UNFPA. | Relevant to SRHR and gender-transformative programming, it does not constitute a training intervention or curriculum for healthcare workers. Instead, it functions as a strategic and operational guidance document |
| Kenya Legal & Ethical Issues Network on HIV and AIDS (KELIN). (2023). Training curriculum for the advancement of sexual and reproductive health rights in the East Africa region. KELIN. | Curriculum targets SRHR advocates and civil-society actors rather than healthcare workers, and does not provide training content for clinical providers. |
| Kingdom of Swaziland, Ministry of Health. Adolescent Sexual and Reproductive Health: Health Sector National Guidelines. Mbabane: Ministry of Health; 2013. Published with support from the United Nations Population Fund (UNFPA). | A national health sector guideline, not a training curriculum or educational program for healthcare workers |
| Levack, A., Rolleri, L. A., DeAtley, J., Trevino, N., & Colbert, M. (2020). Gender Matters: A gender-transformative sexual health curriculum (2nd ed.). New York, NY: EngenderHealth. ISBN 978-1-937410-27-8. | Designed for adolescents/youth, not healthcare workers delivering SRHR services |
| Ministry of Health (MoH), Republic of Uganda. (2017). The national policy guidelines and service standards for sexual and reproductive health and rights (4th ed.). Reproductive Health Division, Department of Community Health. | A national SRHR policy and service standards document that guides service delivery and health system organization, not a training intervention |
| Ministry of Health (Rwanda), Jhpiego, & USAID Maternal and Child Survival Program (MCSP). (2017). Gender and quality of care: Health provider focus group discussion guide. Kigali, Rwanda: MCSP | A research/data collection tool (FGD guide) intended to elicit provider perspectives on gender and quality of care |
| Ministry of Health–AIDS Control Programme (ACP). (2007). HIV counselling supervision: Trainers’ manual (1st ed.). Ministry of Health. | Focuses entirely on HIV counseling supervision (clinical supervision skills), not SRHR, and no bias-reflection or values-clarification content. Target includes supervisors, not SRHR HCWs. |
| Ministry of Health–AIDS Control Programme (ACP). (2007). Positive prevention counselling: A training course for HIV/AIDS counselors— Trainer’s manual (1st ed.). Ministry of Health. | Not an SRHR-training manual for HCWs. Focuses on HIV prevention counseling messages and behavior change, and does not include values clarification or bias-reflection. |
| Ministry of Health, Republic of Liberia. (2021). Adolescent Sexual and Reproductive Health Core Competencies for Health Workers. Monrovia: Ministry of Health. | Guidance and facilitation tool for programme design and reflection |
| Ministry of Health, Uganda. (2006). The national policy guidelines and service standards for sexual and reproductive health and rights (3rd ed.). Reproductive Health Division, Department of Community Health, Ministry of Health, Uganda. | A national policy and service standards guideline |
| Ministry of Health (Uganda), Reproductive Health Unit. (2018). Training of health workers on prevention and management of sexual and gender- based violence and violence against children: Trainee manual. Ministry of Health, Republic of Uganda. | Primarily a clinical and procedural training manual focused on rights- based management of S/GBV, without structured values clarification or reflective components designed to address health workers’ personal beliefs, biases, or gender norms. |
| Population Council. (2009). It’s All One Curriculum: Guidelines and Activities for a Unified Approach to Sexuality, Gender, HIV, and Human Rights Education. New York: Population Council. ISBN 978-0-87834- 117-7 | A comprehensive sexuality education curriculum designed primarily for educators and young people |
| Program for Appropriate Technology in Health (PATH). (2003). Youth- friendly pharmacy program implementation kit: Guidelines and tools for implementing a youth-friendly reproductive health pharmacy program. PATH. | Clinical guidance only; does not contain training modules, structured educational content, or values/bias reflection. |
| Republic of Namibia, Ministry of Health and Social Services. Clinical handbook on the health care of survivors subjected to intimate partner violence and/or sexual violence, Namibia. Windhoek: Ministry of Health and Social Services; 2016. Revised 2021. | A clinical reference handbook providing guidance for clinical management |
| Royal Thai Government, Department of Corrections, Ministry of Justice. (2008). Training of trainers manual on HIV prevention and care in closed settings: Basic level for prison staff. Supported by FHI 360. | Designed for prison guards, not HCWs; does not address SRHR, gender-equitable care, or provider bias. Focus is HIV facts, communication skills, and prison safety. |
| Save the Children & United Nations Population Fund (UNFPA). (2009). Adolescent Sexual and Reproductive Health Toolkit for Humanitarian Settings: A Companion to the Inter-Agency Field Manual on Reproductive Health in Humanitarian Settings. New York: UNFPA & Save the Children. | An operational guidance and implementation directives for agencies and programme managers on what actions should be taken to make ASRH and MISP implementation adolescent-inclusive in humanitarian settings. |
| Schmidt, R., Wolfson, L., Stinson, J., Poole, N., & Greaves, L. (2019). Mothering and Opioids: Addressing Stigma and Acting Collaboratively. Vancouver, BC: Centre of Excellence for Women’s Health. | Not SRHR-focused; targets substance-use and child-welfare practitioners, not SRHR providers. Scope is opioid-use stigma, not FP/abortion/GBV/ASRH. |
| United Nations Population Fund (2022). My Body, My Life, My World Operational Guidance. Module 1: Adolescent Sexual and Reproductive Health and Rights | An operational guidance / programme implementation guidance, not a training intervention or curriculum for healthcare workers. |
| United Nations Population Fund (2022). My Body, My Life, My World Operational Guidance. Module 2: Gender-Based Violence | An operational guidance / programme implementation guidance, not a training intervention or curriculum for healthcare workers. |
| United Nations Population Fund (2025). Resilient Futures: Integrating Sexual and Reproductive Health and Rights, Gender Equality and National Climate Action – A Training Manual for Government and Non- State Actors. Johannesburg: United Nations Population Fund | A training manual, but not for healthcare workers. Designed for government and non-state actors to integrate SRHR into climate policy, and not for HCW competency-building or values reflection. |
| United Nations Population Fund (UNFPA) & Center for Reproductive Rights. Toolkit for advancing human rights-based universal sexual and reproductive health. New York: UNFPA; 2013 | A policy and accountability toolkit, not a training manual or curriculum for healthcare workers. |
| United Nations Population Fund (UNFPA) & Family Health International (FHI). (2008). Family planning and reproductive health: Training manual for providers of youth-friendly services. Cairo, Egypt: UNFPA Egypt and Family Health International. | Technical SRH training only; no values clarification or provider bias/gender-norms components. |
| United Nations Population Fund (UNFPA) & International Confederation of Midwives (ICM). (2011). Comprehensive midwifery programme guidance. UNFPA. | strategic guidance/framework document, not a specific training material |
| United Nations Population Fund (UNFPA) & United Nations Children’s Fund (UNICEF). (2021). Gender-Transformative Accelerator: Niger Country Report. UNFPA–UNICEF Global Programme to End Child Marriage. GTA-Niger-2021 | Not a training material or curriculum designed to build competencies of healthcare workers in SRHR service delivery. Instead, it functions as a programmatic reflection, planning, and assessment tool for UNFPA/UNICEF staff and partners |
| United Nations Population Fund (UNFPA) & United Nations Children’s Fund (UNICEF). (2020). Technical note on gender norms. UNFPA & UNICEF. | A guidance/technical note |
| United Nations Population Fund (UNFPA) & United Nations Children’s Fund (UNICEF). (2021). Gender-Transformative Accelerator Tool: Workshop Facilitation Guide. New York: UNFPA & UNICEF. | Guidance and facilitation tool for programme design and reflection |
| United Nations Population Fund (UNFPA), & Center for Reproductive Rights. (n.d.). Toolkit for advancing human rights-based universal sexual and reproductive health. UNFPA; Center for Reproductive Rights. | A policy/advocacy toolkit for advancing SRHR rights-based approaches. Target users are policymakers and advocates, not healthcare providers. No HCW training component. |
| United Nations Population Fund (UNFPA), & United Nations Children’s Fund (UNICEF). (2024). Advancing gender-transformative approaches in the UNFPA–UNICEF Joint Programme for the Elimination of Female Genital Mutilation: Exploring gender, power, and agency—Integrated Gender-Transformative Accelerator and values clarification workshop facilitation guide (GTA+). UNFPA & UNICEF. | Wrong target population / insufficient healthcare-worker-specific focus: the guide is a broader programme-level, gender-transformative facilitation tool rather than an educational intervention specifically designed to strengthen healthcare workers' competencies for SRHR service provision. |
| United Nations Population Fund (UNFPA), & Women Against Violence Europe (WAVE). (2014). Strengthening health system responses to gender-based violence in Eastern Europe and Central Asia: A resource package. UNFPA Regional Office for Eastern Europe and Central Asia. | A resource package/guidance report on strengthening GBV responses and not a HCW training curriculum |
| United Nations Population Fund (UNFPA). (2010). Health sector response to gender-based violence: An assessment of the Asia Pacific region. UNFPA Asia and the Pacific Regional Office. | A guidance document for UN program planning, not a training curriculum or HCW-focused educational intervention. No structured training or SRHR clinical content. |
| United Nations Population Fund (UNFPA). (2014). UNFPA Operational Guidance for Comprehensive Sexuality Education: A Focus on Human Rights and Gender. New York: UNFPA. | An operational guidance for Comprehensive Sexuality Education (CSE) focused primarily on children, adolescents, teachers, educators, policymakers, and programme managers |
| United Nations Population Fund (UNFPA). (2022). Guidance note: Integrating the gender and social norms output into country programme documents. New York: UNFPA. | A strategic and programmatic guidance, not a training or educational material for healthcare workers |
| United Nations Population Fund (UNFPA). (2022). UNFPA Strategy for Family Planning 2022–2030. New York: UNFPA. | A strategic policy framework rather than a competency-based training material or educational intervention for healthcare workers. |
| United Nations Population Fund (UNFPA). (2023). Gender- Transformative Accelerator (GTA): Workshop Facilitation Guide. UNFPA. | A programming/guidance document/technical note |
| United Nations Population Fund (UNFPA). Supplement to background paper on universal access to sexual and reproductive health and rights as an essential element of universal health coverage. New York: UNFPA; 2019. | A policy/technical background paper. |
| United Nations Population Fund (UNFPA). Women and young persons with disabilities: Guidelines for providing rights-based and gender- responsive services to address gender-based violence and sexual and reproductive health and rights. New York: UNFPA; 2018. | A guideline, No training component |
| Women’s Global Network for Reproductive Rights (WGNRR) Africa. (n.d.). Youth sexual and reproductive health and rights (SRHR) training toolkit: Building champions’ skills on gender, sexuality, SRHR and human rights. WGNRR Africa. | Target audience is youth, not healthcare workers. Does not build SRHR provider competencies. |
| Women’s Refugee Commission, & Save the Children. (2012). Adolescent sexual and reproductive health programs in humanitarian settings: An in- depth look at family planning services. Women’s Refugee Commission. | A research and programme mapping report that documents ASRH service delivery models and good practices |
| World Health Organization (WHO), United Nations Population Fund (UNFPA), and United Nations High Commissioner for Refugees (UNHCR). (2018). Inter-agency Field Manual on Reproductive Health in Humanitarian Settings. Geneva: WHO. | Guidance/toolkit (programmatic directives) |
| World Health Organization. Core competencies in adolescent health and development for primary care providers: including a tool to assess the adolescent health and development component in pre-service education of health-care providers. Geneva: World Health Organization; 2015. ISBN: 978-92-4-150831-5. | Attitudes and rights are referenced, but no explicit values clarification, bias reduction, or gender-norm transformation intervention is described. |
| World Health Organization, Department of Child and Adolescent Health and Development. (2010). Adolescent job aid: A handy desk reference tool for primary level health workers. World Health Organization. | A clinical reference handbook, not a training curriculum. Provides guidance but no training program, no values-clarification, no bias-reflection exercises. |
| World Health Organization. (2013). Midwifery educator core competencies. World Health Organization. | Competency framework for educators, not a training intervention. No HCW sessions, no SRHR biases, no values-clarification. |
| World Health Organization. (2013). Responding to intimate partner violence and sexual violence against women: WHO clinical and policy guidelines. World Health Organization | A clinical and policy guideline, not a discrete training intervention |
| World Health Organization. (2023). Our bodies, our rights! An in-person workshop on addressing sexual and reproductive health and rights and gender-based violence for women and young people with disabilities. World Health Organization. | strongly rights-based and gender-responsive, but it is designed for women and young people with disabilities (community empowerment) rather than healthcare workers delivering SRHR services |
| AWHONN, the Association of Women’s Health, Obstetric and Neonatal Nurses | does not include a specific training materials or programs for HCW related to SRHR |
| Arena 2024 | Assessment/reflection tool only |
| Austad 2017 | Focuses on patient support rather than training healthcare workers in SRHR or addressing provider biases. |
| Austen 2015 | A clinical/procedural, not aimed at SRHR competencies or values clarification. |
| Bohren 2020 | does not include a specific training materials or programs for HCW related to SRHR |
| Brown 2023 | Not an intervention study |
| Campbell 2005 | A narrative review of Sexual Assault Nurse Examiner (SANE) programs focusing on survivor, medical, legal, and community outcomes |
| Cooper 2023 | Educational interventions on affirming care for sexual and gender minorities; A systematic review |
| Crable 2013 | it targets residential care staff and not HCWs; does not focus on SRHR service delivery. |
| David 2025 | Training does not address gender bias or gender norms |
| Denno 2021 | Does not address SRHR or values-clarification training; A systematic review |
| Dhakal 2022 | Conference proceeding; Details of intervention not reported |
| Duchesne 2021 | Does not address SRHR or values-clarification training; A systematic review |
| DuMont 2017 | Training does not address gender bias or gender norms |
| Effland 2018 | Training does not address gender bias or gender norms |
| Flick 2025 | Does not address SRHR topics; It focuses on perinatal opioid/substance use |
| Fontein-Kuipers 2018 | Training does not address SRHR bias reduction |
| Foss 2020 | Does not address SRHR topics or include values clarification or bias- reduction related to SRHR |
| Foster 2024 | Implementation article with insufficient description of the educational intervention |
| Geiser 2020 | Gender bias in clinical practice (general); does not address SRHR content domain |
| Geibel 2017 | Focus on HIV prevention and associated stigma |
| Grayson 2025 | Does NOT meet SRHR content criteria and did not explicitly include reflection on biases/values or rights-based care. |
| Heidarian 2025 | A systematic review; trainings did not include values/bias/norm reflection or rights-based care |
| Heitzler 2012 | Conference proceeding; Details of intervention not reported |
| Hornor 2017 | Clinical indications, laboratory methods, and diagnostic protocols for STI testing |
| Howard 2017 | Does not address SRHR topics; It focuses on perinatal opioid/substance use |
| Islam 2006 | A clinical obstetric and anesthesia skill-based training |
| Iwata 2012 | Does not address SRHR topics or include values clarification or bias- reduction related to SRHR |
| Kane 2024 | Knowledge-focused training with partial attention to respectful care |
| Keedle 2023 | Focuses on interprofessional education and collaboration skill, not value clarification, bias reflection, or SRHR care |
| Keuroghlian 2021 | Commentary article |
| Kutner 2020 | Has to do with HIV services |
| Lalonde 2021 | Editorial |
| Lelutiu-Weinberger 2023 | Focuses on LGBTQ-affirmative mental health care, not on SRHR services or topics |
| Lindhardt 2014 | Motivational interviewing skills training for obstetric providers on weight-management counselling; no gender norms/bias content and no structured values clarification or reflective component targeting providers' own beliefs |
| Maga 2024 | Does not address SRHR topics or promotes reflection on biases/values/norms |
| Mannava 2024 | An informational abortion-law toolkit, not a training or educational intervention |
| Maquibar 2018 | Does not focus on reducing gender bias in SRH |
| Mason 2013 | Preceptorship program focused on general professional development |
| McDowell 2020 | Discusses educational strategies but is not a formal training program, curriculum, or instructional tool |
| McMahon 2018 | Commentary describing programmatic experiences promoting respectful maternal care, without a clearly defined training or educational intervention |
| Mihaly 2022 | Focused on clinical skill only |
| Montano 2022 | Focused on students’ experiences in clinical practice |
| Mwakawanga 2023 | Does not meet SRHR content criteria and did not explicitly include reflection on biases/values or rights-based care |
| Muwanguzi 2023 | Focus on HIV prevention and associated stigma |
| Nielson 2015 | Did not implement a training intervention; examines associations between prior SANE training and provider attitudes |
| Noonan 2024 | Does not address SRHR topics or include values clarification or bias- reduction related to SRHR |
| Norouzi 2019 | Focused on clinical skill only |
| Norris 2024 | Did not implement a training intervention |
| Nugroho 2019 | Focused on HIV/STI outreach, communication, teamwork, and SOGIE awarenessF |
| Patchen 2024 | Describes a multifaceted trauma-informed perinatal care program; No discrete training curriculum/interveniton described |
| Plowright 2018 | A research study analyzing ethical guidelines |
| Pratt-Chapman 2020 | does not include a specific training materials or programs for HCW related to SRHR |
| Royce 2023 | A literature review of cultural competence |
| Scannell 2014 | Focused on operational scheduling strategies and not values clarification or SRHR content |
| Sedlecky 2020 | A survey of national educational practice |
| Shroff 2018 | Appears to focus on clinical skill; Intervention details not provided |
| Simon 2026 | Intervention was implemented as a quality improvement initiative rather than a discrete, structured training curriculum or educational program for HCWs |
| Stephenson 2024 | Procedural training focused on screening practices and implementation |
| Streed 2019 | Intervention details not provided |
| Stueben 2024 | Assessed a single scenario from an existing toolkit; Not a full training program, manual, or curriculum. |
| Suttle 2025 | No training material, toolkit, curriculum, or educational program for HCWs related to SRHR |
| Turan 2022 | Does not focus on SRH service delivery or SRHR-specific care domain |
| Tyson 2016 | A licensure and competency assessment, not on strengthening SRHR- related reflective competencies or addressing bias in SRHR care |
| VanBorek 2025 | Advocacy video, not a training |
| Vijayalakshmi 2021 | Focused on abused among women with mental illness in mental health setting |
| Walker 2016 | discusses educational strategies but is not a formal training program, curriculum, or instructional tool |
| Walsh 2025 | Vignettes used for assessment only |
| Young 2020 | No specific training program/material/manual. It is a literature review of cultural competence |
| Zbikowski 2012 | An analysis of ethical guidelines/code, not a training intervention/tools for healthcare workers. |

# References to Excluded Interventional Studies

1. AWHONN, the Association of Women’s Health, Obstetric and Neonatal Nurses (2021). Racism and Bias in Maternity Care Settings. *Nursing for Women’s Health*, *25*(5), e1–e3. <https://doi.org/10.1016/j.nwh.2021.06.002>
2. Arena, F., Geiser, E., Auer, S., & Clair, C. (2024). Reflexivity and positionality applied to medical practice: A study on implicit gender bias with medical students in a Swiss university. *… Journal for Equity in Health*. <https://doi.org/10.1186/s12939-024-02222-3>
3. Austad, K., Chary, A., Martinez, B., Juarez, M., Martin, Y. J., Ixen, E. C., & Rohloff, P. (2017). Obstetric care navigation: A new approach to promote respectful maternity care and overcome barriers to safe motherhood. *Reproductive Health*, *14*(1), 148. <https://doi.org/10.1186/s12978-017-0410-6>
4. Auten, J. D., Ross, E. M., French, M. A., Li, I. Z., Robinson, L., Brown, N., King, K. J., & Tanen, D. A. (2015). Low-fidelity hybrid sexual assault simulation training’s effect on the comfort and competency of resident physicians. *The Journal of Emergency Medicine*, *48*(3), 344–350. <https://doi.org/10.1016/j.jemermed.2014.09.032>
5. Bohren, M. A., Tunçalp, Ö., & Miller, S. (2020). Transforming intrapartum care: Respectful maternity care. *Best Practice & Research. Clinical Obstetrics & Gynaecology*, *67*, 113–126. <https://doi.org/10.1016/j.bpobgyn.2020.02.005>
6. Brown, M., McCann, E., McLoughlin, G., Martin, C. H., & McCormick, F. (2023). The views and experiences of midwifery academics regarding LGBTQ+ health education in pre-registration programmes in the United Kingdom and Ireland: Qualitative findings from a mixed-methods study. *Nurse Education in Practice*, *67*(101090848), 103554. <https://doi.org/10.1016/j.nepr.2023.103554>
7. Campbell, R., Patterson, D., & Lichty, L. F. (2005). The effectiveness of sexual assault nurse examiner (SANE) programs: A review of psychological, medical, legal, and community outcomes. *Trauma, Violence & Abuse*, *6*(4), 313–329. <https://doi.org/10.1177/1524838005280328>
8. Cooper, R. L., Ramesh, A., Radix, A. E., & Reuben, J. S. (2023). Affirming and inclusive care training for medical students and residents to reduce health disparities experienced by sexual and gender minorities: A systematic review. *… Health*. <https://doi.org/10.1089/trgh.2021.0148>
9. Crable, A. R., Underwood, L. A., Parks-Savage, A., & Maclin, V. (2013). An Examination of a Gender-Specific and Trauma-Informed Training Curriculum: Implications for Providers. *International Journal of Behavioral Consultation and Therapy*, *7*(4), 30–37.
10. David, J.-C., Rascle, N., Auriacombe, M., Serre, F., Sutter-Dallay, A.-L., & Loyal, D. (2025). A video-based intervention to overcome pregnancy smoking stigma among healthcare students: A randomised controlled trial. *Psychology & Health*, *40*(8), 1303–1322. <https://doi.org/10.1080/08870446.2024.2316677>
11. Denno, D. M., & Plesons, M. (2021). Effective strategies to improve health worker performance in delivering adolescent-friendly sexual and reproductive health services. *… Medicine and Health*. <https://doi.org/10.1515/ijamh-2019-0245>
12. Dhakal, M. P., Creedy, D., Gamble, J., Newnham, E., & McInnes, R. (n.d.). P33—Effects of an online education intervention on nursing students’ perceptions towards respectful maternity care...Australian College of Midwives National Conference—Together at the Top, September 13-15, 2022, Cairns, Queensland, Australia. *Women & Birth*, *35*, N.PAG-N.PAG. <https://doi.org/10.1016/j.wombi.2022.07.148>
13. Du Mont J., Kosa D., Macdonald S., & Mason R. (2017). The promise of an interactive, online curriculum in improving the competence of those working in healthcare settings to address sexual assault. *Journal of Multidisciplinary Healthcare*, *10*((Du Mont, Mason) Women’s College Research Institute, Women’s College Hospital, Canada(Du Mont, Mason) Dalla Lana School of Public Health, University of Toronto, Canada(Kosa, Macdonald) Ontario Network of Sexual Assault/Domestic Violence Treatment Centres,), 425EP – 427. <https://doi.org/10.2147/JMDH.S148075>
14. Duchesne, S., Donnadieu, A.-C., Chariot, P., & Louis-Sylvestre, C. (2021). Screening for domestic violence during pregnancy follow-up: Evaluation of an intervention in an antenatal service. *Archives of Women’s Mental Health*, *24*(2), 293–301. <https://doi.org/10.1007/s00737-020-01058-4>
15. Effland, K. J., & Hays, K. (2018). A web-based resource for promoting equity in midwifery education and training: Towards meaningful diversity and inclusion. *Midwifery*, *61*(8510930, mwf), 70–73. <https://doi.org/10.1016/j.midw.2018.02.008>
16. Flick, S. A. (2025). Trauma-informed care education for the perinatal nurse: A quality improvement project. *Dissertation Abstracts International: Section B: The Sciences and Engineering*, *86*(12-B), No-Specified.
17. Fontein-Kuipers, Y., & Romeijn, E. (2018). ISeeYou—Evaluation of a woman-centred care pilot project in Bachelor midwifery education and research. *Midwifery*, *58*(8510930, mwf), 1–5. <https://doi.org/10.1016/j.midw.2017.11.005>
18. Foss, M., le May, W. A., & Gobbi, M. (2020). The development and testing of a co-designed distance learning programme to improve the knowledge of trained auxiliary nurse midwives in normal midwifery practice in central India. *Journal of Research in Nursing : JRN*, *25*(6–7), 541–558. <https://doi.org/10.1177/1744987120952836>
19. Foster W., Williams N., Wood J., Dyer S., & Minkus A. (2024). Adaptation and Implementation of the RNAO woman abuse best practice guideline: A critical reflection. *MedUNAB*, *27*(1), 32EP – 41. <https://doi.org/10.29375/01237047.4652>
20. Geiser E., Clair C., Auer S., Carrier J.-M., Fasel E., & Schwarz J. (2020). Reflexivity during medical internships: How to think about gender in clinical practice. *Revue Medicale Suisse*, *16*(713), 2104EP – 2107. <https://doi.org/10.53738/REVMED.2020.16.713.2104>
21. Geibel, S., Hossain, S. M. I., Pulerwitz, J., Sultana, N., Hossain, T., Roy, S., Burnett-Zieman, B., Stackpool-Moore, L., Friedland, B. A., Yasmin, R., Sadiq, N., & Yam, E. (2017). Stigma Reduction Training Improves Healthcare Provider Attitudes Toward, and Experiences of, Young Marginalized People in Bangladesh. *The Journal of Adolescent Health : Official Publication of the Society for Adolescent Medicine*, *60*(2S2), S35–S44. <https://doi.org/10.1016/j.jadohealth.2016.09.026>
22. Grayson, N. D., Quinones, N., King, K., Norman, K., Oseguera, T. W., Smith, N., Peake, M., & Amore, A. D. (2025). The CHOICES Black Midwifery Fellowship: An Innovative Model for Training Midwives in Reproductive Justice and Community-Centered Care. *Journal of Midwifery & Women’s Health*, *dml, 100909407*. <https://doi.org/10.1111/jmwh.70028>
23. Heidarian, H., Mehrabi, M., Ghaemmaghami, P., & Janghorban, R. (2025). Effect of E-learning clinical management of substance-dependent pregnant women on the knowledge and clinical skill performance of midwives: A randomized controlled trial. *BMC Pregnancy and Childbirth*, *25*(1), 11. <https://doi.org/10.1186/s12884-024-07130-6>
24. Heitzler, E. T. (2012). An Innovative Approach to Increasing the Cultural Competence of Nurses Who Care for Childbearing Women and Newborns. *JOGNN: Journal of Obstetric, Gynecologic & Neonatal Nursing*, *41*, S161–S161. <https://doi.org/10.1111/j.1552-6909.2012.01362_60.x>
25. Hornor, G. (2017). Sexually Transmitted Infections and Children: What the PNP Should Know. *Journal of Pediatric Health Care : Official Publication of National Association of Pediatric Nurse Associates & Practitioners*, *31*(2), 222–229. <https://doi.org/10.1016/j.pedhc.2016.04.016>
26. Howard H. & Clark K. (2017). Interprofessional-shared decision making for pregnant women with opioid use: Results from a provider training. *Current Women’s Health Reviews*, *13*(2), 121EP – 129. <https://doi.org/10.2174/1573404813666170511115024>
27. Islam, M. T., Haque, Y. A., Waxman, R., & Bhuiyan, A. B. (2006). Implementation of emergency obstetric care training in Bangladesh: Lessons learned. *Reproductive Health Matters*, *14*(27), 61–72. <https://doi.org/10.1016/S0968-8080(06)27229-X>
28. Iwata, H., Mori, E., Morita, A., Sakajo, A., Maekawa, T., Ozawa, H., & Maehara, K. (2012). Evaluation of the training course for a nursing intervention programme to promote maternal role attainment in pregnant Japanese women who have undergone assisted reproductive technology. *International Journal of Nursing Practice (John Wiley & Sons, Inc.)*, *18*(Supp S2), 45–55. <https://doi.org/10.1111/j.1440-172X.2012.02028.x>
29. Kane, D., Kennedy, K. M., Flood, K., & Eogan, M. (2024). General practice trainees’ understanding of post-sexual assault care: The impact of a specialist educational intervention. *Irish Journal of Medical Science*, *193*(3), 1331–1336. <https://doi.org/10.1007/s11845-023-03576-3>
30. Keedle, H., Stulz, V., Conti, J., Bentley, R., Meade, T., Qummouh, R., Hay, P., Kaye-Smith, H., Everitt, L., & Schmied, V. (2023). Psychosocial interprofessional perinatal education: Design and evaluation of an interprofessional learning experience to improve students’ collaboration skills in perinatal mental health. *Women and Birth : Journal of the Australian College of Midwives*, *36*(4), e379–e387. <https://doi.org/10.1016/j.wombi.2023.01.001>
31. Keuroghlian, A. S., & Mujugira, A. (2021). Healthcare worker training to improve quality of care for sexual and gender minority people in sub‐Saharan Africa: Learning from efforts in Uganda. *Journal of the …*. <https://doi.org/10.1002/jia2.25728>
32. Kutner, B. A., Wu, Y., Balan, I. C., & Meyers, K. (2020). “Talking About it Publicly Made Me Feel Both Curious and Embarrassed”: Acceptability, Feasibility, and Appropriateness of a Stigma-Mitigation Training to Increase Health Worker Comfort Discussing Anal Sexuality in HIV Services. *AIDS and Behavior*, *24*(6), 1951–1965. <https://doi.org/10.1007/s10461-019-02758-4>
33. Lalonde A.B., Herschderfer K., & Miller S. (2021). FIGO collaboration for safe and respectful maternity care. *International Journal of Gynecology and Obstetrics*, *152*(3), 285EP – 287. <https://doi.org/10.1002/ijgo.13310>
34. Lelutiu-Weinberger, C., Clark, K. A., & Pachankis, J. E. (2023). Mental Health Provider Training to Improve LGBTQ Competence and Reduce Implicit and Explicit Bias: A Randomized Controlled Trial of Online and In-Person Delivery. *Psychology of Sexual Orientation and Gender Diversity*, *10*(4), 589–599. <https://doi.org/10.1037/sgd0000560>
35. Lindhardt, C. L., Rubak, S., Mogensen, O., Hansen, H. P., Lamont, R. F., & Jorgensen, J. S. (2014). Training in motivational interviewing in obstetrics: A quantitative analytical tool. *Acta Obstetricia et Gynecologica Scandinavica*, *93*(7), 698–704. <https://doi.org/10.1111/aogs.12401>
36. Maga, G., Magon, A., Caruso, R., Brigante, L., Daniele, M. A. S., Belloni, S., & Arrigoni, C. (2024). Development and Validation of the Midwifery Interventions Classification for a Salutogenic Approach to Maternity Care: A Delphi Study. *Healthcare (Basel, Switzerland)*, *12*(22). <https://doi.org/10.3390/healthcare12222228>
37. Mannava S.V., Brar A., Patwardhan U., Godfrey J., Berdan E., Gow K., & Knod J.L. (2024). Bridging the Knowledge Gap: A Toolkit on Reproductive Rights for Pediatric Surgeons in the Post-Dobbs Era. *Journal of Pediatric Surgery*, *59*(7), 1374EP – 1377. <https://doi.org/10.1016/j.jpedsurg.2024.03.029>
38. Maquibar, A., Hurtig, A.-K., Vives-Cases, C., Estalella, I., & Goicolea, I. (2018). Nursing students’ discourses on gender-based violence and their training for a comprehensive healthcare response: A qualitative study. *Nurse Education Today*, *68*(ned, 8511379), 208–212. <https://doi.org/10.1016/j.nedt.2018.06.011>
39. Mason, J., & Davies, S. (2013). A qualitative evaluation of a preceptorship programme to support newly qualified midwives. *Evidence Based Midwifery*, *11*(3), 94–98.
40. McDowell, M. J., Goldhammer, H., & Potter, J. E. (2020). Strategies to mitigate clinician implicit bias against sexual and gender minority patients. *Psychosomatics*. <https://www.sciencedirect.com/science/article/pii/S0033318220301523>
41. McMahon, S. A., Mnzava, R. J., Tibaijuka, G., & Currie, S. (2018). The “hot potato” topic: Challenges and facilitators to promoting respectful maternal care within a broader health intervention in Tanzania. *Reproductive Health*, *15*(1), 153. <https://doi.org/10.1186/s12978-018-0589-1>
42. Mihaly, L. K., & Gatewood, E. (2022). A Workshop To Develop Nurse Practitioner Competencies in Women’s Reproductive Health. *Journal for Nurse Practitioners*, *18*(7), 761–764. <https://doi.org/10.1016/j.nurpra.2022.02.007>
43. Montano, M., Macias, V., Molina, R. L., Aristizabal, P., & Nigenda, G. (2022). The experience of obstetric nursing students in an innovative maternal care programme in Chiapas, Mexico: A qualitative study. *Sexual and Reproductive Health Matters*, *30*(1), 2095708. <https://doi.org/10.1080/26410397.2022.2095708>
44. Muwanguzi, P. A., Nabunya, R., Karis, V. M. S., Nabisere, A., Nangendo, J., & Mujugira, A. (2023). Nurses’ reflections on caring for sexual and gender minorities pre-post stigma reduction training in Uganda. *BMC Nursing*, *22*(1), 50. <https://doi.org/10.1186/s12912-023-01208-w>
45. Mwakawanga, D. L., Rimoy, M., Mwanga, F., Massae, A. F., Mushy, S. E., Kisaka, L., Komba, N., Mabada, L., Mlay, E., Mwakalinga, E., Mwasha, L., Temba, F. F., & Sirili, N. (2023). Strengthening midwives’ competencies for addressing maternal and newborn mortality in Tanzania: Lessons from Midwifery Emergency Skills Training (MEST) project. *Midwifery*, *122*(8510930, mwf), 103695. <https://doi.org/10.1016/j.midw.2023.103695>
46. Nielson, M. H., Strong, L., & Stewart, J. G. (2015). Does Sexual Assault Nurse Examiner (SANE) Training Affect Attitudes of Emergency Department Nurses Toward Sexual Assault Survivors?. *Journal of Forensic Nursing*, *11*(3), 137–143. <https://doi.org/10.1097/JFN.0000000000000081>
47. Noonan, M., Brown, M., Gibbons, M., Tuohy, T., Johnson, K., Bradshaw, C., Tighe, S. M., Atkinson, S., Murphy, L., Mohamad, M., Imcha, M., O’Dwyer, N., & Grealish, A. (2024). Evaluation of the effectiveness of a video-based educational intervention on perinatal mental health related stigma reduction strategies for healthcare professionals: A single group pre-test-post-test pilot study. *Midwifery*, *136*(8510930, mwf), 104089. <https://doi.org/10.1016/j.midw.2024.104089>
48. Norouzi, Z., Jafarnejad, F., Khadivzadeh, T., Esmaily, H., & Hedjazi, A. (2019). Comparison of the effect of standardized patient-based training with team-based learning on the knowledge of midwifery students in providing services to victims of rape. *Journal of Education and Health Promotion*, *8*(101593794), 267. <https://doi.org/10.4103/jehp.jehp_237_18>
49. Norris, G., Hollins Martin, C. J., Moylan, A., & Greig, Y. (2024). A qualitative descriptive training needs analysis of midwives perceived continuous professional development in providing intranatal respectful maternal care. *Nurse Education Today*, *136*(ned, 8511379), 106144. <https://doi.org/10.1016/j.nedt.2024.106144>
50. Nugroho, A., Coulter, R. W. S., Erasmus, V., Laksmono, P. A., Mihari, T. S., & Richardus, J. H. (2019). Evaluation of a training aimed at building capacity for outreaching to men who have sex with men and transgender women in Indonesia. *Health Education Research*, *34*(2), 223–233. <https://doi.org/10.1093/her/cyy046>
51. Patchen, L., McCullers, A., Beach, C., Browning, M., Porter, S., Danielson, A., Asegieme, E., Richardson, S. R., Jost, A., Jensen, C. S., & Ahmed, N. (2024). Safe Babies, Safe Moms: A Multifaceted, Trauma Informed Care Initiative. *Maternal & Child Health Journal*, *28*(1), 31–37. <https://doi.org/10.1007/s10995-023-03840-z>
52. Plowright, A., Taylor, C., Davies, D., Sartori, J., Hundt, G. L., & Lilford, R. J. (2018). Formative evaluation of a training intervention for community health workers in South Africa: A before and after study. *PloS One*, *13*(9), e0202817. <https://doi.org/10.1371/journal.pone.0202817>
53. Pratt-Chapman, M. L. (2020). Implementation of sexual and gender minority health curricula in health care professional schools: A qualitative study. *BMC Medical Education*. <https://doi.org/10.1186/s12909-020-02045-0>
54. Royce, C. S., Morgan, H. K., Baecher-Lind, L., Cox, S., Everett, E. N., Fleming, A., Graziano, S. C., Sims, S. M., Morosky, C., Sutton, J., & Sonn, T. (2023). The time is now: Addressing implicit bias in obstetrics and gynecology education. *American Journal of Obstetrics and Gynecology*, *228*(4), 369–381. <https://doi.org/10.1016/j.ajog.2022.12.016>
55. Scannell, M. (2014). Scheduling Strategy Using Poisson Probability to Ensure 24/7 Scheduling Coverage for Emergency Nurses Caring for Patients that Have Been Sexually Assaulted. *Journal of Midwifery & Women’s Health*, *59*(5), 550–551. <https://doi.org/10.1111/jmwh.12242>
56. Sedlecky, K., Rašević, M., & Bitzer, J. (2020). Education and training of health care workers for contraceptive service delivery in 21 countries across Europe. *Sexual & Reproductive HealthCare*, *24*, N.PAG-N.PAG. <https://doi.org/10.1016/j.srhc.2020.100498>
57. Shroff, S., Spataro, B., Jeong, K., Rothenberger, S., Rubio, D., & McNeil, M. (2018). Let’s talk about sex: Development and evaluation of a sexual history and counseling curriculum for internal medicine interns. *Patient Education and Counseling*, *101*(7), 1298–1301. <https://doi.org/10.1016/j.pec.2018.02.016>
58. Simon, N. (2026). Provider mindfulness: Changing implicit bias and improving perinatal health outcomes for patients with adverse social determinants of health. *Dissertation Abstracts International: Section B: The Sciences and Engineering*, *87*(1-B), No-Specified.
59. Stephenson, L. (2024). Nurse readiness in screening for intimate partner violence in the perinatal setting. *Dissertation Abstracts International: Section B: The Sciences and Engineering*, *85*(11-B), No-Specified.
60. Streed, C. G. J., Hedian, H. F., Bertram, A., & Sisson, S. D. (2019). Assessment of Internal Medicine Resident Preparedness to Care for Lesbian, Gay, Bisexual, Transgender, and Queer/Questioning Patients. *Journal of General Internal Medicine*, *34*(6), 893–898. <https://doi.org/10.1007/s11606-019-04855-5>
61. Stueben, F., Beacom, R., Larson, A., & Simon, P. (n.d.). Implementing an LGBTQ+ interprofessional simulation with undergraduate nursing students. *International Journal of Nursing Education Scholarship*, *20*(1). <https://doi.org/10.1515/ijnes-2024-0057>
62. Suttle, B., Bordelon, C., Jones, C., Talarico, N., Nguyen, S., & Bryant, P. (2025). Improving Care for LGBTQ+ Families: A Competency Bundle. *Journal of Perinatal & Neonatal Nursing*, *39*(2), 111–117. <https://doi.org/10.1097/JPN.0000000000000888>
63. Turan, F. D. (2022). Effects of a structured online educational program course on nursing students’ attitudes toward gender roles and women and children’s violence abuse reports: A quasi-experimental evaluation. *Nurse Education Today*, *108*(ned, 8511379), 105191. <https://doi.org/10.1016/j.nedt.2021.105191>
64. Tyson H. & Wilson-Mitchell K. (2016). Diversifying the Midwifery Workforce: Inclusivity, Culturally Sensitive Bridging, and Innovation. *Journal of Midwifery and Women’s Health*, *61*(6), 752EP – 758. <https://doi.org/10.1111/jmwh.12573>
65. Van Borek, S., Logie, C. H., Mbende, B., Ghoma Linguissi, L. S., MacKenzie, F., Ouedraogo, A., Lad, A., Boumba, A., Gittings, L., & Loemba, H. (2025). Esengo ya Bosembo (“Joy of Equity”): Development of an Advocacy Video to Reduce Stigma and to Promote Sexual and Reproductive Health and Rights of Women Sex Professionals in Pointe-Noire, Congo Republic. *Health Promotion Practice*, *26*(6), 1052–1058. <https://doi.org/10.1177/15248399241245053>
66. Vijayalakshmi, P., Sailaxmi, G., Nikhil Reddy, S. S., Marimuthu, P., & Suresh, B. M. (2021). Effectiveness of a Module Based Training on Nurses’ Attitude Towards Social Norms and Beliefs That Support Abuse Among women with Mental Illness. *Community Mental Health Journal*, *57*(1), 161–166. <https://doi.org/10.1007/s10597-020-00628-1>
67. Walker, K., Arbour, M., & Waryold, J. (2016). Educational Strategies to Help Students Provide Respectful Sexual and Reproductive Health Care for Lesbian, Gay, Bisexual, and Transgender Persons. *Journal of Midwifery & Women’s Health*, *61*(6), 737–743. <https://doi.org/10.1111/jmwh.12506>
68. Walsh, K., Klein, L. B., Luebke, J., Moore, K. M., Ruiz, A. M., Curran, K., & Melnik, J. (2025). Sexual Assault Nurse Examiner’s Confidence to Care for Black, Indigenous, and Transgender Survivors: Development and Preliminary Content Validity of Practice Vignettes. *Journal of Emergency Nursing*, *7605913*. <https://doi.org/10.1016/j.jen.2024.12.005>
69. Young, S., & Guo, K. L. (2020). Cultural diversity training: The necessity of cultural competence for health care providers and in nursing practice. *The Health Care Manager*. <https://journals.lww.com/healthcaremanagerjournal/fulltext/2020/04000/Cultural_Diversity_Training__The_Necessity_of.7.aspx>
70. Zbikowski, A., Bruggemann, A. J., Wijma, B., Zeiler, K., & Swahnberg, K. (2012). Ethical guidelines and the prevention of abuse in healthcare. *European Journal of Obstetrics, Gynecology, and Reproductive Biology*, *165*(1), 18–28. <https://doi.org/10.1016/j.ejogrb.2012.07.018> (N.d.).
