# Supplementary material for: Addressing gender bias in sexual and reproductive health care: A scoping review of training tools and interventions
Source: PLoS One. 2026 Oct 13;21(10):e0359704. doi: 10.1371/journal.pone.0359704 (PMC13660924; doi:10.1371/journal.pone.0359704)
Supplement: Supplementary file 1 [file pone.0359704.s001.zip › Supporting Information S1 - 5/S3 File Supplementary Codebook.docx]

# S3 File: Supplementary Codebook

Each included source was independently coded using the decision rules below. Binary variables were coded as **1 (present)** or **0 (absent)** based on explicit descriptions in the source. Where classifications (e.g., gender-transformative elements, delivery modality, content domains, transformative focus, or level of influence) required interpretation, coding was based on the stated objectives, curriculum content, and intervention activities reported in the source. Ambiguous cases were resolved through discussion among reviewers until consensus was reached. Multiple categories could be assigned for non-mutually exclusive variables where explicitly described.

| **Variable** | **Coding definition / decision rule** |
| --- | --- |
| **Experiential Learning** | Code as "1" if the intervention describes active or applied learning (e.g., simulation, role-play, case/scenario discussions, skills practice, participatory exercises, drama, vignettes, video-based exercises, standardized patients, or other experiential activities). Otherwise, code as "0". |
| **Reflective or Values-Based** | Code as "1" if the intervention includes values clarification, VCAT, reflection, journaling, bias/stigma reduction, attitude exploration, empathy-building, self-awareness, or other structured reflective learning. Otherwise, code as "0". |
| **Didactic Instruction** | Code as "1" if lectures, classroom teaching, seminars, presentations, modules, or other didactic instructional approaches are described. Otherwise, code as "0". |
| **Digital or Online** | Code as "1" if any component is delivered online, web-based, mobile, virtual, asynchronous, AI-supported, virtual reality, e-learning, or through blended digital delivery. Otherwise, code as "0". |
| **Reinforcement or Implementation Support** | Code as "1" if the intervention includes mentorship, coaching, supervision, follow-up sessions, quality improvement activities, peer support, action planning, workplace projects, refresher training, or implementation support beyond the initial training. Otherwise, code as "0". |
| **Equitable or Rights-Based Care** | Code as "1" if the intervention includes rights-based, equity-oriented, person-centred, client-centred, survivor-centred, respectful, inclusive, or culturally responsive care. Otherwise, code as "0". |
| **Gender Transformative Elements** | Code as "1" when the intervention addresses gender norms, unequal power relations, structural gender inequality, intersectionality, feminist approaches, anti-racism, or gender-transformative change. Otherwise, code as "0". |
| **System Level Elements** | Code as **1** if organizational, institutional, leadership, policy, accountability, referral, workflow, multisectoral, or quality improvement components are included; Otherwise, code as "0". |
| **Content Domains** | Assign one or more topical domains according to the primary curriculum content described in the source. |
| **Delivery Modality** | Classify the primary delivery format as In-person**,** Online/digital**,** Blended/mixed**,** Manual/toolkit (delivery adaptable)**,** or Not specified |
| **Pedagogical Methods** | Assign all teaching methods described (e.g., simulation, role-play, case-based learning, participatory learning, skills practice, reflection, VCAT). |
| **Target Level (Level of Influence)** | Assign **all applicable levels of influence** targeted by the intervention. Categories are **not mutually exclusive**. Code one or more of: **Individual:** targets provider knowledge, attitudes, values, skills, or behaviors; **Interpersonal:** targets provider–patient/family communication, relationships, empathy, respectful care, or shared decision-making; **Structural:** targets organizational, institutional, leadership, policy, workflow, accountability, quality improvement, referral systems, or broader health system factors. |
