# Supplementary material for: Addressing gender bias in sexual and reproductive health care: A scoping review of training tools and interventions
Source: PLoS One. 2026 Oct 13;21(10):e0359704. doi: 10.1371/journal.pone.0359704 (PMC13660924; doi:10.1371/journal.pone.0359704)
Supplement: Supplementary file 1 [file pone.0359704.s001.zip › Supporting Information S1 - 5/S1 File Search strategy.docx]

# **S1 File: Search strategy**

**CINAHL Database Search Details**

Date of Search: 20 November 2025

Total Number of Records Retrieved – 1814

**Search Strategy**

1. (MH "Gender Equality")
2. (MH "Gender Role")
3. (MH Sexism)
4. (MH "Women's Rights")
5. (MH Feminism)
6. (MH "Gender-Based Violence")
7. XB (sexism* OR sexist* OR feminism* OR feminist*)
8. XB ((gender OR gendered OR genderi* OR wom#n* OR girl*) N6 (norms OR role OR roles OR bias* OR debias* OR discrimina* OR prejudic* OR prejudis* OR equity OR equitabl* OR equal* OR inequity OR inequitabl* OR inequal* OR unequal* OR right* OR liberation OR status OR fair* OR unfair* OR justice OR injustice OR unjust OR mainstream* OR mistreatment OR neglect OR violence OR harm* OR depriv* OR assault* OR aggression* OR injury OR injuries OR abuse* OR abusive* OR batter* OR disrespect* OR respectful OR dignified OR nondignified OR undignified OR stereotyp* OR stigma* OR marginaliz* OR marginalis* OR disparit* OR sensitiv* OR competenc* OR transformative OR abandon*))
9. XB ((sex* OR female*) N3 (norms OR roles OR bias* OR debias* OR discrimina* OR prejudic* OR prejudis* OR equity OR equitabl* OR equal* OR inequity OR inequitabl* OR inequal* OR unequal* OR right* OR liberation OR fair* OR unfair* OR justice OR injustice OR unjust OR mainstream* OR mistreatment OR neglect OR violence OR depriv* OR assault* OR aggression* OR abuse* OR abusive* OR batter* OR disrespect* OR respectful OR dignified OR nondignified OR undignified OR stereotyp* OR stigma* OR marginaliz* OR marginalis* OR competenc* OR transformative OR abandon*))
10. XB ("wom#n centered" OR "wom#n centred" OR "wom#n oriented" OR "female* centered" OR "female* centred" OR "female* oriented" OR "sex* disparit*" OR "wom#n disparit*" OR "female* disparit*" OR "sex* sensitiv*" OR "wom#n sensitiv*" OR "female* sensitiv*")
11. XB ((maternal OR maternity OR obstetric* OR gyn#ecolog* OR pregnan* OR lactat* OR breastfeed* OR "breast feed*" OR birth OR childbirth OR stillbirth* OR abortion* OR miscarriage* OR midwife* OR midwive* OR doula* OR prenatal* OR antenatal* OR neonat* OR perinatal* OR postnatal* OR antepartum OR postpartum OR post-partum OR ante-partum OR post-natal* OR pre-natal* OR ante-natal* OR neo-nat* OR peri-natal* OR gestation* OR newborn* OR "new born*" OR fetal OR foetal OR fetus OR foetus) N3 (bias* OR debias* OR discrimina* OR prejudic* OR prejudis* OR equity OR equitabl* OR equal* OR inequity OR inequitabl* OR inequal* OR unequal* OR right* OR fair* OR unfair* OR justice OR injustice OR unjust OR mistreatment OR neglect OR violence OR depriv* OR assault* OR aggression* OR abuse* OR abusive* OR batter* OR disrespect* OR respectful OR dignified OR nondignified OR undignified OR stereotyp* OR stigma* OR marginaliz* OR marginalis* OR abandon*))
12. S1 OR S2 OR S3 OR S4 OR S5 OR S6 OR S7 OR S8 OR S9 OR S10 OR S11
13. (MH "reproductive health") OR (MH "sexual health") OR (MH "women's health+") OR (MH "maternal-child health") OR (MH "Family Planning+") OR (MH contraception)
14. (MH gynecology) OR (MH obstetrics)
15. (MH "Alternative birth centers") OR (MH "fertility clinics") OR (MH "obstetric service")
16. (MH "Diagnosis, Ob-Gyn")
17. (MH lactation+) OR (MH "perinatal period") OR (MH "postnatal period+") OR (MH "pregnancy trimesters") OR (MH pregnancy+) OR (MH "labor+") OR (MH parturition)
18. (MH "maternal health services+") OR (MH "perinatal care") OR (MH "postnatal care") OR (MH "prenatal care")
19. (MH "pregnancy complications+") OR (MH "abortion, spontaneous") OR (MH "diabetes mellitus, gestational") OR (MH "perinatal death") OR (MH "fetal diseases") OR (MH "pregnancy-induced hypertension") OR (MH pre-eclampsia) OR (MH "labor complications+") OR (MH "childbirth, premature") OR (MH "postpartum hemorrhage") OR (MH "placenta diseases") OR (MH "puerperal disorders") OR (MH "depression, postpartum") OR (MH "lactation disorders")
20. (MH "surgery, obstetrical+") OR (MH "delivery, obstetric+") OR (MH "cesarean section") OR (MH "surgery, gynecologic+") OR (MH hysterectomy)
21. ((TI maternal) OR (TI maternity) OR (TI obstetric*) OR (TI gyn#ecolog*) OR (TI pregnan*) OR (TI lactat*) OR (TI breastfeed*) OR (TI "breast feed*") OR (TI birth*) OR (TI childbirth*) OR (TI stillbirth*) OR (TI abortion*) OR (TI miscarriage*) OR (TI midwife*) OR (TI midwive*) OR (TI doula*) OR (TI prenatal*) OR (TI antenatal*) OR (TI neonat*) OR (TI perinatal*) OR (TI postnatal*) OR (TI antepartum) OR (TI postpartum) OR (TI post-partum) OR (TI ante-partum) OR (TI post-natal*) OR (TI pre-natal*) OR (TI ante-natal*) OR (TI neo-nat*) OR (TI peri-natal*) OR (TI gestation*) OR (TI newborn*) OR (TI "new born*") OR (TI fetal) OR (TI foetal) OR (TI fetus) OR (TI foetus) OR (TI eclampsia*) OR (TI parturition) OR (TI placenta*) OR (TI puerperal) OR (TI puerperium) OR (TI cesarean) OR (TI caesarean) OR (TI cesarian) OR (TI caesarian) OR (TI cesarien) OR (TI caesarien))
22. XB ((reproductive OR sexual*) N4 (health* OR care OR medicine OR service* OR right*)) OR ((TI SRH) OR (TI SRHR))
23. XB (wom#n* N2 (health* OR right*))
24. ((TI "family planning") OR (TI contracepti*) OR (TI "birth control"))
25. S13 OR S14 OR S15 OR S16 OR S17 OR S18 OR S19 OR S20 OR S21 OR S22 OR S23 OR S24
26. (MH "health personnel") OR (MH "allied health personnel") OR (MH "community health workers") OR (MH "emergency medical technicians") OR (MH "certified nursing assistants") OR (MH "physician assistants") OR (MH anesthetists+) OR (MH doulas) OR (MH "health educators") OR (MH "medical staff+") OR (MH "medical staff, hospital+") OR (MH nurses+) OR (MH "certified nurse midwives") OR (MH "staff nurses+") OR (MH "nursing staff, hospital") OR (MH "personnel, health facility") OR (MH physicians+) OR (MH gynecologists) OR (MH obstetricians) OR (MH "health facility administrators+")
27. (MH "health occupations") OR (MH "allied health Professions") OR (MH "health services administration") OR (MH "health facility administration") OR (MH “nursing as a profession”) OR (MH "registered nursing practice+") OR (MH "practical nursing")
28. ((TI physician*) OR (TI doctor*) OR (TI gyn#ecologist*) OR (TI obstetrician*) OR (TI nurse*) OR (TI nursing) OR (TI practitioner*) OR (TI "health* staff") OR (TI "health* worker*") OR (TI "health* personnel") OR (TI "health* technician*") OR (TI "health* assistant*") OR (TI "health* aide*") OR (TI "health* officer*") OR (TI "health care staff") OR (TI "health care worker*") OR (TI "health care personnel") OR (TI "health care technician*") OR (TI "health care assistant*") OR (TI "health care aide*") OR (TI "health care officer*") OR (TI "medical staff") OR (TI "medical worker*") OR (TI "medical personnel") OR (TI "medical technician*") OR (TI "medical assistant*") OR (TI "medical aide*") OR (TI "medical officer*") OR (TI "hospital staff") OR (TI "hospital worker*") OR (TI "hospital personnel") OR (TI "hospital technician*") OR (TI "hospital assistant*") OR (TI "hospital aide*") OR (TI "hospital officer*") OR (TI midwife*) OR (TI midwives) OR (TI doula*) OR (TI "outreach worker*") OR (TI lay) OR (TI frontline) OR (TI counselor*) OR (TI counsellor*) OR (TI coach*) OR (TI "health* promoter*") OR (TI "health care promoter*") OR (TI "health* extension worker*") OR (TI "health care extension worker*") OR (TI "health* provider*") OR (TI "health* attendant*") OR (TI "health care provider*") OR (TI "health care attendant*") OR (TI "medical provider*") OR (TI "medical attendant*") OR (TI "birth* attendant*") OR (TI companion*) OR (TI paraprofessional*) OR (TI intern*) OR (TI resident*) OR (TI clinician*) OR (TI attendee*) OR (TI trainee*) OR (TI apprentice*) OR (TI workforce) OR (TI "health* professional*") OR (TI "health care professional*”) OR (TI “medical professional*") OR (TI "social worker*") OR (TI policymaker*) OR (TI "policy maker*") OR (TI "health* manager*") OR (TI "health care manager*") OR (TI "hospital manager*"))
29. S26 OR S27 OR S28
30. S12 AND S25 AND S29
31. (MH animals+)
32. (MH “animal studies”)
33. (TI “animal model*”)
34. S31 OR S32 OR S33
35. (MH human)
36. S34 NOT S35
37. S30 NOT S36
38. ((PT editorial) OR (PT comment) OR (PT news) OR (PT "newspaper article"))
39. S37 NOT S38
40. (MH education) OR (MH curriculum+) OR (MH "education, continuing+") OR (MH "education, medical+") OR (MH "education, nursing+") OR (MH "professional competence") OR (MH "clinical competence") OR (MH "staff development") OR (MH mentorship) OR (MH teaching)
41. (MH Health policy)
42. (MH "Quality Improvement")
43. (MH "quality assurance")
44. (MH "codes of ethics")
45. (MH "practice guidelines")
46. (MH "teaching materials+")
47. (MH "Training Support, financial") OR (MH "Internship and Residency") OR (MH "Remedial Teaching") OR (MH "Patient Rounds")
48. (MH "health education")
49. ((TI quality) N3 ((TI improv*) OR (TI assurance) OR (TI indicator*)))
50. ((TI educat*) OR (TI curricul*) OR (TI competence) OR (TI inservice*) OR (TI train*) OR (TI development) OR (TI mentor*) OR (TI teach*) OR (TI rounds))
51. ((TI course*) OR (TI workshop*) OR (TI seminar*) OR (TI lecture*) OR (TI handout*) OR (TI manual*) OR (TI book) OR (TI books) OR (TI textbook*) OR (TI class) OR (TI classes) OR (TI learn*) OR (TI practicum) OR (TI campaign*))
52. ((TI program*) OR (TI policy) OR (TI policies) OR (TI rule) OR (TI rules) OR (TI implement*) OR (TI intervention*) OR (TI guide*) OR (TI guidance) OR (TI tool*) OR (TI approach*) OR (TI strategy) OR (TI strategies))
53. S40 OR S41 OR S42 OR S43 OR S44 OR S45 OR S46 OR S47 OR S48 OR S49 OR S50 OR S51 OR S52
54. S39 AND S53
55. "limit 54 to Publication date="2000 -Current""

**GOOGLE SCHOLAR SEARCH DETAILS**

**Summary of Search Output**

**Date of Search: 16 November 2025**

Total No. of Studies Retrieved – 800

Duplicate Records Removed in Endnote (Prior to import into Covidence) – 124

Total Number of Records Imported into Covidence – 676

**Search Strategy (Search Strings)**

(Gender AROUND (2) (norms|Bias*|Discrimination|transformative|Competenc*)) (“Health Worker*”|”Healthcare Worker*”|Doctor*|Nurse*|Midwi*|Physician*) (“Reproduct* Health”|SRH|SRHR|Sexual AROUND (3) Health) (Training|Tool*|Educat*|Intervention*|Approach*)

(Gender AROUND (2) (norms|Bias*|Discrimination|transformative|Competenc*)) (“social worker*”|“policy maker*”|“health * manager*”)) (“Reproduct* Health”|SRH|SRHR|Sexual AROUND (3) Health) (Training|Tool*|Educat*|Intervention*|Approach*)

(Gender AROUND (2) (norms|Bias*|Discrimination|transformative|Competenc*)) (“Health Worker*”|”Healthcare Worker*”|Doctor*|Nurse*|Midwi*|Physician*) (“Reproduct* Health”|SRH|SRHR|Sexual AROUND (3) Health) (Guide*|Policies|policy|program*|Manual*|Curricul*)

(Gender AROUND (2) (norms|Bias*|Discrimination|transformative|Competenc*)) (“social worker*”|“policy maker*”|“health * manager*”) (“Reproduct* Health”|SRH*|Sexual AROUND (3) Health) (Guide*|Policies|policy|program*|Manual*|Curricul*)

**Organizational WebsitesDate of Search: 03 November 2025**

Total No. of Pages Retrieved – 27

Number of toolkits/manuals/curriculums - 270

Searched google scholar “"training manual" OR "toolkit" OR "curriculum") ("gender norms" OR "gender bias" OR "values clarification" OR "gender transformative") ("sexual and reproductive health" OR "SRHR") site:who.int OR site:unfpa.org OR site:ippf.org OR site:jhpiego.org OR site:ipas.org OR site:path.org OR site:engenderhealth.org”

**Ovid multi-databases: Embase Classic+Embase 1947 to 2025 November 14; Ovid MEDLINE(R) ALL 1946 to November 17, 2025; APA PsycInfo 1806 to November 2025 Week 2; EBM Reviews - Cochrane Central Register of Controlled Trials October 2025; ERIC 1965 to October 2025**

Date of Search: November 17, 2025

Total Number of Records Retrieved – 5001

1.	Gender Equity/

2.	Gender Role/

3.	Sexism/

4.	Women's Rights/ or reproductive rights/

5.	Feminism/

6.	Gender-Based Violence/

7.	(sexism* or sexist* or feminism* or feminist*).mp.

8.	((gender or gendered or genderi* or wom?n* or girl*) adj6 (norms or role or roles or bias* or debias* or discrimina* or prejudic* or prejudis* or equity or equitabl* or equal* or inequity or inequitabl* or inequal* or unequal* or right* or liberation or status or fair* or unfair* or justice or injustice or unjust or mainstream* or mistreatment or neglect or violence or harm* or depriv* or assault* or aggression* or injury or injuries or abuse* or abusive* or batter* or disrespect* or respectful or dignified or nondignified or undignified or stereotyp* or stigma* or marginaliz* or marginalis* or disparit* or sensitiv* or competenc* or transformative or abandon* or ethic*)).mp.

9.	((sex* or female*) adj3 (norms or roles or bias* or debias* or discrimina* or prejudic* or prejudis* or equity or equitabl* or equal* or inequity or inequitabl* or inequal* or unequal* or right* or liberation or fair* or unfair* or justice or injustice or unjust or mainstream* or mistreatment or neglect or violence or depriv* or assault* or aggression* or abuse* or abusive* or batter* or disrespect* or respectful or dignified or nondignified or undignified or stereotyp* or stigma* or marginaliz* or marginalis* or competenc* or transformative or abandon* or ethic*)).mp.

10.	(wom?n centered or wom?n centred or wom?n oriented or female* centered or female* centred or female* oriented or sex* disparit* or wom?n disparit* or female* disparit* or sex* sensitiv* or wom?n sensitiv* or female* sensitiv*).mp.

11.	((maternal or maternity or obstetric* or gyn?ecolog* or pregnan* or lactat* or breastfeed* or breast feed* or birth or childbirth or stillbirth* or abortion* or miscarriage* or midwife* or midwive* or doula* or prenatal* or antenatal* or neonat* or perinatal* or postnatal* or antepartum or postpartum or post-partum or ante-partum or post-natal* or pre-natal* or ante-natal* or neo-nat* or peri-natal* or gestation* or newborn* or new born* or fetal or foetal or fetus or foetus) adj3 (bias* or debias* or discrimina* or prejudic* or prejudis* or equity or equitabl* or equal* or inequity or inequitabl* or inequal* or unequal* or right* or fair* or unfair* or justice or injustice or unjust or mistreatment or neglect or violence or depriv* or assault* or aggression* or abuse* or abusive* or batter* or disrespect* or respectful or dignified or nondignified or undignified or stereotyp* or stigma* or marginaliz* or marginalis* or abandon* or ethic*)).mp.

12.	1 or 2 or 3 or 4 or 5 or 6 or 7 or 8 or 9 or 10 or 11

13.	reproductive health/ or sexual health/ or exp women's health/ or maternal health/ or exp reproductive health services/ or Family Planning/ or Family Planning Services/ or contraception/

14.	reproductive medicine/ or gynecology/ or obstetrics/

15.	maternal-child health centers/ or birthing centers/ or fertility clinics/ or "obstetrics and gynecology department, hospital"/

16.	exp "Diagnostic Techniques, Obstetrical and Gynecological"/

17.	exp lactation/ or peripartum period/ or exp postpartum period/ or pregnancy trimesters/ or exp pregnancy/ or exp labor, obstetric/ or parturition/

18.	exp maternal health services/ or perinatal care/ or postnatal care/ or prenatal care/

19.	exp pregnancy complications/ or abortion, spontaneous/ or diabetes, gestational/ or fetal death/ or stillbirth/ or fetal diseases/ or hypertension, pregnancy-induced/ or pre-eclampsia/ or exp obstetric labor complications/ or premature birth/ or postpartum hemorrhage/ or placenta diseases/ or puerperal disorders/ or depression, postpartum/ or lactation disorders/

20.	exp obstetric surgical procedures/ or exp delivery, obstetric/ or cesarean section/ or exp gynecologic surgical procedures/ or hysterectomy/

21.	(maternal or maternity or obstetric* or gyn?ecolog* or pregnan* or lactat* or breastfeed* or breast feed* or birth* or childbirth* or stillbirth* or abortion* or miscarriage* or midwife* or midwive* or doula* or prenatal* or antenatal* or neonat* or perinatal* or postnatal* or antepartum or postpartum or post-partum or ante-partum or post-natal* or pre-natal* or ante-natal* or neo-nat* or peri-natal* or gestation* or newborn* or new born* or fetal or foetal or fetus or foetus or eclampsia* or parturition or placenta* or puerperal or puerperium or cesarean or caesarean or cesarian or caesarian or cesarien or caesarien).ti,kf,kw.

22.	((reproductive or sexual*) adj6 (health* or care or medicine or service* or right*)).mp. or (SRH or SRHR).ti.

23.	(wom?n* adj2 (health* or right*)).mp.

24.	(family planning or contracepti* or birth control).ti,kf,kw.

25.	13 or 14 or 15 or 16 or 17 or 18 or 19 or 20 or 21 or 22 or 23 or 24

26.	health personnel/ or allied health personnel/ or community health workers/ or emergency medical technicians/ or nursing assistants/ or physician assistants/ or exp anesthetists/ or doulas/ or health educators/ or exp medical staff/ or exp medical staff, hospital/ or exp nurses/ or nurse midwives/ or exp nursing staff/ or nursing staff, hospital/ or personnel, hospital/ or exp physicians/ or gynecologists/ or obstetricians/ or exp health facility administrators/ or hospital administrators/

27.	health occupations/ or allied health occupations/ or health services administration/ or hospital administration/ or nursing/ or exp specialties, nursing/ or nursing, practical/

28.	(physician* or doctor* or gyn?ecologist* or obstetrician* or nurse* or nursing or practitioner* or health* staff or health* worker* or health* personnel or health* technician* or health* assistant* or health* aide* or health* officer* or health care staff or health care worker* or health care personnel or health care technician* or health care assistant* or health care aide* or health care officer* or medical staff or medical worker* or medical personnel or medical technician* or medical assistant* or medical aide* or medical officer* or hospital staff or hospital worker* or hospital personnel or hospital technician* or hospital assistant* or hospital aide* or hospital officer* or midwife* or midwives or doula* or outreach worker* or lay or frontline or counselor* or counsellor* or coach* or health* promoter* or health care promoter* or health* extension worker* or health care extension worker* or health* provider* or health* attendant* or health care provider* or health care attendant* or medical provider* or medical attendant* or birth* attendant* or companion* or paraprofessional* or intern* or resident* or clinician* or attendee* or trainee* or apprentice* or workforce or health* professional* or health care professional* or medical professional* or social worker* or policymaker* or policy maker* or health* manager* or health care manager* or hospital manager*).ti,kf,kw.

29.	26 or 27 or 28

30.	12 and 25 and 29

31.	exp animals/ not humans/

32.	30 not 31

33.	(editorial or comment or news or newspaper article).pt. or comment/ or editorial/ or news/ or comment*.ti.

34.	32 not 33

35.	limit 34 to yr="2000 -Current"

36.	education/ or exp curriculum/ or exp education, professional/ or exp education, continuing/ or exp education, medical/ or exp education, nursing/ or professional competence/ or clinical competence/ or inservice training/ or staff development/ or mentoring/ or teaching/

37.	policy/

38.	Quality Improvement/

39.	quality assurance, health care/

40.	exp guidelines as topic/ or "codes of ethics"/ or practice guidelines as topic/

41.	exp guideline/ or practice guideline/

42.	exp teaching materials/ or manuals as topic/

43.	Training Support/ or "Internship and Residency"/ or Remedial Teaching/

44.	health education/

45.	*human rights/ or *patient rights/ or *informed consent/

46.	(quality adj3 (improvement or assurance or indicator* or management)).ti,kf,kw.

47.	(educat* or curricul* or competence or inservice* or in service* or train* or development or mentor* or teach*).ti,kf,kw.

48.	(course* or workshop* or seminar* or lecture* or handout* or manual* or book or books or textbook* or class or classes or learn* or practicum or campaign*).ti,kf,kw.

49.	(program* or policy or policies or rule or rules or implement* or intervention* or guide* or guidance or tool*).ti,kf,kw.

50.	36 or 37 or 38 or 39 or 40 or 41 or 42 or 43 or 44 or 45 or 46 or 47 or 48 or 49

51.	35 and 50

52.	limit 35 to ("scoping review" or "systematic review")

53.	limit 35 to (guideline or practice guideline or study guide)

54.	51 or 52 or 53

55.	54 use medall

56.	Gender Equity/

57.	*sex role/

58.	exp sexism/ or exp gender based violence/ or gender bias/

59.	*Women's Rights/ or reproductive rights/

60.	Feminism/

61.	(sexism* or sexist* or feminism* or feminist*).mp.

62.	((gender or gendered or genderi* or wom?n* or girl*) adj4 (norms or role or roles or bias* or debias* or discrimina* or prejudic* or prejudis* or equity or equitabl* or equal* or inequity or inequitabl* or inequal* or unequal* or right* or liberation or status or fair* or unfair* or justice or injustice or unjust or mainstream* or mistreatment or neglect or violence or harm* or depriv* or assault* or aggression* or injury or injuries or abuse* or abusive* or batter* or disrespect* or respectful or dignified or nondignified or undignified or stereotyp* or stigma* or marginaliz* or marginalis* or disparit* or sensitiv* or competenc* or transformative or abandon* or ethic*)).mp.

63.	((sex* or female*) adj3 (norms or roles or bias* or debias* or discrimina* or prejudic* or prejudis* or equity or equitabl* or equal* or inequity or inequitabl* or inequal* or unequal* or right* or liberation or fair* or unfair* or justice or injustice or unjust or mainstream* or mistreatment or neglect or violence or depriv* or assault* or aggression* or abuse* or abusive* or batter* or disrespect* or respectful or dignified or nondignified or undignified or stereotyp* or stigma* or marginaliz* or marginalis* or competenc* or transformative or abandon* or ethic*)).ti,kf,kw.

64.	(wom?n centered or wom?n centred or wom?n oriented or female* centered or female* centred or female* oriented).mp.

65.	((maternal or maternity or obstetric* or gyn?ecolog* or pregnan* or lactat* or breastfeed* or breast feed* or birth or childbirth or stillbirth* or abortion* or miscarriage* or midwife* or midwive* or doula* or prenatal* or antenatal* or neonat* or perinatal* or postnatal* or antepartum or postpartum or post-partum or ante-partum or post-natal* or pre-natal* or ante-natal* or neo-nat* or peri-natal* or gestation* or newborn* or new born* or fetal or foetal or fetus or foetus) adj4 (bias* or debias* or discrimina* or prejudic* or prejudis* or equity or equitabl* or equal* or inequity or inequitabl* or inequal* or unequal* or right* or fair* or unfair* or justice or injustice or unjust or mistreatment or neglect or violence or depriv* or assault* or aggression* or abuse* or abusive* or batter* or disrespect* or respectful or dignified or nondignified or undignified or stereotyp* or stigma* or marginaliz* or marginalis* or abandon* or ethic*)).ti,kf,kw.

66.	56 or 57 or 58 or 59 or 60 or 61 or 62 or 63 or 64 or 65

67.	reproductive health/

68.	exp sexual health/

69.	*women's health/

70.	exp maternal care/ or exp perinatal care/ or maternal child health care/ or maternal welfare/

71.	exp birth control/ or exp contraception/ or exp family planning/ or exp induced abortion/

72.	exp labor/ or exp childbirth/ or labor management/

73.	gynecology/ or obstetrics/

74.	maternity ward/

75.	gynecologic disease/

76.	exp pregnancy/

77.	exp abortion/ or exp spontaneous abortion/

78.	lactation/

79.	perinatal period/ or prenatal period/

80.	maternal health service/

81.	exp obstetric procedure/ or exp obstetric delivery/ or exp perinatal care/ or exp postnatal care/ or exp prenatal care/

82.	exp pregnancy complication/ or pregnancy disorder/ or exp maternal disease/

83.	exp gynecologic surgery/ or hysterectomy/

84.	(maternal or maternity or obstetric* or gyn?ecolog* or pregnan* or lactat* or breastfeed* or breast feed* or birth* or childbirth* or stillbirth* or abortion* or miscarriage* or midwife* or midwive* or doula* or prenatal* or antenatal* or neonat* or perinatal* or postnatal* or antepartum or postpartum or post-partum or ante-partum or post-natal* or pre-natal* or ante-natal* or neo-nat* or peri-natal* or gestation* or newborn* or new born* or fetal or foetal or fetus or foetus or eclampsia* or parturition or placenta* or puerperal or puerperium or cesarean or caesarean or cesarian or caesarian or cesarien or caesarien).ti,kf,kw.

85.	((reproductive or sexual*) adj4 (health* or care or medicine or service* or right*)).mp. or (SRH or SRHR).ti.

86.	(wom?n* adj2 health*).ti,kf,kw. or (wom?n* adj2 right*).mp.

87.	(family planning or contracepti* or birth control).ti,kf,kw.

88.	67 or 68 or 69 or 70 or 71 or 72 or 73 or 74 or 75 or 76 or 77 or 78 or 79 or 80 or 81 or 82 or 83 or 84 or 85 or 86 or 87

89.	exp medical personnel/ or medical specialist/ or medical staff/ or exp physician/ or resident/

90.	exp nurse/ or nurse administrator/

91.	exp *nursing/

92.	paramedical personnel/ or doula/ or *health practitioner/ or health visitor/ or lactation consultant/ or exp midwife/ or traditional birth attendant/ or *nursing assistant/ or *nursing staff/

93.	health care personnel/ or exp anesthesist/ or *clinician/ or *health auxiliary/ or *health workforce/ or lay health worker/ or *advanced practice provider/ or *physician assistant/

94.	*hospital personnel/ or *hospital administrator/

95.	*medical profession/

96.	*paramedical profession/

97.	(physician* or doctor* or gyn?ecologist* or obstetrician* or nurse* or nursing or practitioner* or health* staff or health* worker* or health* personnel or health* technician* or health* assistant* or health* aide* or health* officer* or health care staff or health care worker* or health care personnel or health care technician* or health care assistant* or health care aide* or health care officer* or medical staff or medical worker* or medical personnel or medical technician* or medical assistant* or medical aide* or medical officer* or hospital staff or hospital worker* or hospital personnel or hospital technician* or hospital assistant* or hospital aide* or hospital officer* or midwife* or midwives or doula* or outreach worker* or lay or frontline or counselor* or counsellor* or coach* or health* promoter* or health care promoter* or health* extension worker* or health care extension worker* or health* provider* or health* attendant* or health care provider* or health care attendant* or medical provider* or medical attendant* or birth* attendant* or companion* or paraprofessional* or intern* or resident* or clinician* or attendee* or trainee* or apprentice* or workforce or health* professional* or health care professional* or medical professional* or social worker* or policymaker* or policy maker* or health* manager* or health care manager* or hospital manager*).ti,kf,kw.

98.	89 or 90 or 91 or 92 or 93 or 94 or 95 or 96 or 97

99.	66 and 88 and 98

100.	Animal experiment/ not (human experiment/ or human/)

101.	(rodent* or squirrel* or rat or rats or mouse or mice or swine or porcine or murine or sheep or lambs or pigs or piglets or rabbit or rabbits or cat or cats or dog or dogs or cattle or bovine or trout or marmoset$1 or primate*).ti. and animal experiment/

102.	editorial/ or editorial.pt. or note/ or note.pt. or comment/ or comment*.ti.

103.	100 or 101 or 102

104.	99 not 103

105.	limit 104 to yr="2000 -Current"

106.	*education/ or continuing education/ or course content/ or curriculum/ or curriculum development/ or education program/ or exp educational tool/ or in service training/ or exp medical education/ or exp mentoring/ or exp paramedical education/ or exp nursing education/ or teaching/ or educational material/

107.	*vocational education/

108.	policy/ or organizational policy/

109.	exp health care policy/ or public policy/

110.	*total quality management/

111.	exp medical guideline/ or exp practice guideline/ or clinical practice guideline/

112.	*consent/ or *medical ethics/ or exp *informed consent/

113.	*human rights/ or *patient right/

114.	book/

115.	staff training/

116.	standard operating procedure/

117.	(educat* or curricul* or inservice* or in service* or train* or mentor* or teach*).ti,kf,kw.

118.	(course* or workshop* or seminar* or lecture* or handout* or manual* or book or books or textbook* or class or classes or learn*).ti,kf,kw.

119.	(program* or policy or policies or rule or rules or intervention* or guide* or guidance or tool*).ti,kf,kw.

120.	106 or 107 or 108 or 109 or 110 or 111 or 112 or 113 or 114 or 115 or 116 or 117 or 118 or 119

121.	105 and 120

122.	limit 105 to "systematic review"

123.	121 or 122

124.	limit 123 to embase

125.	124 use emczd

126.	12 or 66

127.	25 or 88

128.	29 or 98

129.	50 or 120

130.	126 and 127 and 128 and 129

131.	limit 130 to yr="2000 -Current"

132.	131 use cctr

133.	gender bias/ or gender discrimination/

134.	sex fairness/ or feminism/

135.	sex role/

136.	(sexism* or sexist* or feminism* or feminist*).mp.

137.	((gender or gendered or genderi* or wom?n* or girl*) adj6 (norms or role or roles or bias* or debias* or discrimina* or prejudic* or prejudis* or equity or equitabl* or equal* or inequity or inequitabl* or inequal* or unequal* or right* or liberation or status or fair* or unfair* or justice or injustice or unjust or mainstream* or mistreatment or neglect or violence or harm* or depriv* or assault* or aggression* or injury or injuries or abuse* or abusive* or batter* or disrespect* or respectful or dignified or nondignified or undignified or stereotyp* or stigma* or marginaliz* or marginalis* or disparit* or sensitiv* or competenc* or transformative or abandon* or ethic*)).mp.

138.	((sex* or female*) adj3 (norms or roles or bias* or debias* or discrimina* or prejudic* or prejudis* or equity or equitabl* or equal* or inequity or inequitabl* or inequal* or unequal* or right* or liberation or fair* or unfair* or justice or injustice or unjust or mainstream* or mistreatment or neglect or violence or depriv* or assault* or aggression* or abuse* or abusive* or batter* or disrespect* or respectful or dignified or nondignified or undignified or stereotyp* or stigma* or marginaliz* or marginalis* or competenc* or transformative or abandon* or ethic*)).mp.

139.	(wom?n centered or wom?n centred or wom?n oriented or female* centered or female* centred or female* oriented or sex* disparit* or wom?n disparit* or female* disparit* or sex* sensitiv* or wom?n sensitiv* or female* sensitiv*).mp.

140.	((maternal or maternity or obstetric* or gyn?ecolog* or pregnan* or lactat* or breastfeed* or breast feed* or birth or childbirth or stillbirth* or abortion* or miscarriage* or midwife* or midwive* or doula* or prenatal* or antenatal* or neonat* or perinatal* or postnatal* or antepartum or postpartum or post-partum or ante-partum or post-natal* or pre-natal* or ante-natal* or neo-nat* or peri-natal* or gestation* or newborn* or new born* or fetal or foetal or fetus or foetus) adj3 (bias* or debias* or discrimina* or prejudic* or prejudis* or equity or equitabl* or equal* or inequity or inequitabl* or inequal* or unequal* or right* or fair* or unfair* or justice or injustice or unjust or mistreatment or neglect or violence or depriv* or assault* or aggression* or abuse* or abusive* or batter* or disrespect* or respectful or dignified or nondignified or undignified or stereotyp* or stigma* or marginaliz* or marginalis* or abandon* or ethic*)).mp.

141.	133 or 134 or 135 or 136 or 137 or 138 or 139 or 140

142.	pregnancy/ or birth/ or contraception/ or family planning/ or gynecology/ or obstetrics/ or prenatal care/

143.	sexually transmitted diseases/

144.	(maternal or maternity or obstetric* or gyn?ecolog* or pregnan* or lactat* or breastfeed* or breast feed* or birth* or childbirth* or stillbirth* or abortion* or miscarriage* or midwife* or midwive* or doula* or prenatal* or antenatal* or neonat* or perinatal* or postnatal* or antepartum or postpartum or post-partum or ante-partum or post-natal* or pre-natal* or ante-natal* or neo-nat* or peri-natal* or gestation* or newborn* or new born* or fetal or foetal or fetus or foetus or eclampsia* or parturition or placenta* or puerperal or puerperium or cesarean or caesarean or cesarian or caesarian or cesarien or caesarien).mp.

145.	((reproductive or sexual*) adj6 (health* or care or medicine or service* or right*)).mp. or (SRH or SRHR).ti.

146.	(wom?n* adj2 (health* or right*)).mp.

147.	(family planning or contracepti* or birth control).mp.

148.	142 or 143 or 144 or 145 or 146 or 147

149.	141 and 148

150.	(physician* or doctor* or gyn?ecologist* or obstetrician* or nurse* or nursing or practitioner* or health* staff or health* worker* or health* personnel or health* technician* or health* assistant* or health* aide* or health* officer* or health care staff or health care worker* or health care personnel or health care technician* or health care assistant* or health care aide* or health care officer* or medical staff or medical worker* or medical personnel or medical technician* or medical assistant* or medical aide* or medical officer* or hospital staff or hospital worker* or hospital personnel or hospital technician* or hospital assistant* or hospital aide* or hospital officer* or midwife* or midwives or doula* or outreach worker* or lay or frontline or counselor* or counsellor* or coach* or health* promoter* or health care promoter* or health* extension worker* or health care extension worker* or health* provider* or health* attendant* or health care provider* or health care attendant* or medical provider* or medical attendant* or birth* attendant* or companion* or paraprofessional* or intern* or resident* or clinician* or attendee* or trainee* or apprentice* or workforce or health* professional* or health care professional* or medical professional* or social worker* or policymaker* or policy maker* or health* manager* or health care manager* or hospital manager*).mp.

151.	exp health personnel/ or exp allied health personnel/ or exp nurses/ or exp physicians/ or exp health occupations/ or allied health occupations/

152.	150 or 151

153.	149 and 152

154.	limit 153 to yr="2000 -Current"

155.	education/ or allied health occupations education/ or inservice education/ or professional education/ or curriculum/ or education courses/ or educational policy/ or educational strategies/ or instruction/ or learning/ or training/

156.	exp continuing education/ or staff development/

157.	educational methods/ or teaching methods/ or training methods/ or educational strategies/ or teaching guides/

158.	exp medical education/ or exp nursing education/ or exp allied health occupations education/

159.	policy/ or educational policy/ or personnel policy/ or school policy/ or policy formation/

160.	quality control/ or quality assurance/ or total quality management/

161.	guidelines/ or guides/

162.	instructional materials/ or educational media/ or study guides/ or teacher developed materials/ or textbooks/ or workbooks/ or curriculum development/ or curriculum implementation/ or educational resources/

163.	program implementation/

164.	ethics/ or consent/ or informed consent/

165.	(quality adj3 (improvement or assurance or indicator* or management or control)).ti,id.

166.	(educat* or curricul* or competence or inservice* or in service* or train* or development or mentor* or teach*).ti,id.

167.	(course* or workshop* or seminar* or lecture* or handout* or manual* or book or books or textbook* or class or classes or learn* or practicum or campaign*).ti,id.

168.	(program* or policy or policies or rule or rules or implement* or intervention* or guide* or guidance or tool*).ti,id.

169.	155 or 156 or 157 or 158 or 159 or 160 or 161 or 162 or 163 or 164 or 165 or 166 or 167 or 168

170.	154 and 169

171.	170 use eric

172.	gender bias/ or gender equality/ or exp gender violence/ or misogyny/ or sex discrimination/ or exp sexism/

173.	sex role attitudes/ or gender role attitudes/ or sex roles/

174.	reproductive rights/ or women's rights/

175.	feminism/

176.	(sexism* or sexist* or feminism* or feminist*).mp.

177.	((gender or gendered or genderi* or wom?n* or girl*) adj6 (norms or role or roles or bias* or debias* or discrimina* or prejudic* or prejudis* or equity or equitabl* or equal* or inequity or inequitabl* or inequal* or unequal* or right* or liberation or status or fair* or unfair* or justice or injustice or unjust or mainstream* or mistreatment or neglect or violence or harm* or depriv* or assault* or aggression* or injury or injuries or abuse* or abusive* or batter* or disrespect* or respectful or dignified or nondignified or undignified or stereotyp* or stigma* or marginaliz* or marginalis* or disparit* or sensitiv* or competenc* or transformative or abandon* or ethic*)).mp.

178.	((sex* or female*) adj3 (norms or roles or bias* or debias* or discrimina* or prejudic* or prejudis* or equity or equitabl* or equal* or inequity or inequitabl* or inequal* or unequal* or right* or liberation or fair* or unfair* or justice or injustice or unjust or mainstream* or mistreatment or neglect or violence or depriv* or assault* or aggression* or abuse* or abusive* or batter* or disrespect* or respectful or dignified or nondignified or undignified or stereotyp* or stigma* or marginaliz* or marginalis* or competenc* or transformative or abandon* or ethic*)).mp.

179.	(wom?n centered or wom?n centred or wom?n oriented or female* centered or female* centred or female* oriented).mp.

180.	((maternal or maternity or obstetric* or gyn?ecolog* or pregnan* or lactat* or breastfeed* or breast feed* or birth or childbirth or stillbirth* or abortion* or miscarriage* or midwife* or midwive* or doula* or prenatal* or antenatal* or neonat* or perinatal* or postnatal* or antepartum or postpartum or post-partum or ante-partum or post-natal* or pre-natal* or ante-natal* or neo-nat* or peri-natal* or gestation* or newborn* or new born* or fetal or foetal or fetus or foetus) adj3 (bias* or debias* or discrimina* or prejudic* or prejudis* or equity or equitabl* or equal* or inequity or inequitabl* or inequal* or unequal* or right* or fair* or unfair* or justice or injustice or unjust or mistreatment or neglect or violence or depriv* or assault* or aggression* or abuse* or abusive* or batter* or disrespect* or respectful or dignified or nondignified or undignified or stereotyp* or stigma* or marginaliz* or marginalis* or abandon* or ethic*)).mp.

181.	172 or 173 or 174 or 175 or 176 or 177 or 178 or 179 or 180

182.	limit 181 to "remove medline records"

183.	exp reproductive health care/ or induced abortion/ or exp prenatal care/ or reproductive health/ or sexual health/

184.	exp family planning/ or exp birth control/

185.	exp obstetrics/ or caesarean birth/ or midwifery/ or gynecology/ or exp obstetrical complications/

186.	exp pregnancy/ or exp pregnancy outcomes/ or exp birth/ or perinatal period/ or postnatal period/ or preeclampsia/ or exp "labor (childbirth)"/

187.	lactation/ or breast feeding/

188.	exp peripartum period/

189.	postpartum depression/

190.	hysterectomy/

191.	183 or 184 or 185 or 186 or 187 or 188 or 189 or 190

192.	182 and 191

193.	(maternal or maternity or obstetric* or gyn?ecolog* or pregnan* or lactat* or breastfeed* or breast feed* or birth* or childbirth* or stillbirth* or abortion* or miscarriage* or midwife* or midwive* or doula* or prenatal* or antenatal* or neonat* or perinatal* or postnatal* or antepartum or postpartum or post-partum or ante-partum or post-natal* or pre-natal* or ante-natal* or neo-nat* or peri-natal* or gestation* or eclampsia* or parturition or placenta* or puerperal or puerperium or cesarean or caesarean or cesarian or caesarian or cesarien or caesarien).ti,id.

194.	((reproductive or sexual*) adj4 (health* or care or medicine or service* or right*)).mp. or (SRH or SRHR).ti.

195.	(family planning or contracepti* or birth control).ti,id.

196.	183 or 184 or 185 or 186 or 187 or 188 or 189 or 190 or 193 or 194 or 195

197.	182 and 196

198.	(physician* or doctor* or gyn?ecologist* or obstetrician* or nurse* or nursing or practitioner* or health* staff or health* worker* or health* personnel or health* technician* or health* assistant* or health* aide* or health* officer* or health care staff or health care worker* or health care personnel or health care technician* or health care assistant* or health care aide* or health care officer* or medical staff or medical worker* or medical personnel or medical technician* or medical assistant* or medical aide* or medical officer* or hospital staff or hospital worker* or hospital personnel or hospital technician* or hospital assistant* or hospital aide* or hospital officer* or midwife* or midwives or doula* or outreach worker* or lay or frontline or counselor* or counsellor* or coach* or health* promoter* or health care promoter* or health* extension worker* or health care extension worker* or health* provider* or health* attendant* or health care provider* or health care attendant* or medical provider* or medical attendant* or birth* attendant* or companion* or paraprofessional* or intern* or resident* or clinician* or attendee* or trainee* or apprentice* or workforce or health* professional* or health care professional* or medical professional* or social worker* or policymaker* or policy maker* or health* manager* or health care manager* or hospital manager*).ti,id,hw,sh.

199.	197 and 198

200.	exp animals/ or exp vertebrates/ or animal models/

201.	exp animal research/

202.	animal.po.

203.	200 or 201 or 202

204.	exp Human Females/ or exp Human Males/

205.	human.po.

206.	204 or 205

207.	203 not 206

208.	199 not 207

209.	(editorial or comment* or news*).ti.

210.	208 not 209

211.	limit 210 to yr="2000 -Current"

212.	(quality adj3 (improvement or assurance or indicator* or management)).ti,id.

213.	(educat* or curricul* or competence or inservice* or in service* or train* or development or mentor* or teach*).ti,id.

214.	(course* or workshop* or seminar* or lecture* or handout* or manual* or book or books or textbook* or class or classes or learn*).ti,id.

215.	(program* or policy or policies or rule or rules or implement* or intervention* or guide* or guidance or tool*).ti,id.

216.	212 or 213 or 214 or 215

217.	211 and 216

218.	217 use psyh

219.	55 or 125 or 132 or 171 or 218

220.	limit 219 to yr="2000 - 2020"

221.	limit 219 to yr="2021 -Current"

222.	220 or 221

223.	remove duplicates from 220

224.	remove duplicates from 221

225.	223 or 224
